# Opportunities and Challenges in the Synthesis of Noble Metal Nanoparticles via the Chemical Route in Microreactor Systems

**DOI:** 10.3390/mi15091119

**Published:** 2024-08-31

**Authors:** Adrianna Pach, Aleksandra Szot, Krzysztof Fitzner, Magdalena Luty-Błocho

**Affiliations:** AGH University of Krakow, Faculty of Non-Ferrous Metals, al. Adama Mickiewicza 30, 30-059 Krakow, Poland; apach@agh.edu.pl (A.P.); szot@agh.edu.pl (A.S.); fitzner@agh.edu.pl (K.F.)

**Keywords:** nanoparticles, microreactor, nucleation and growth, chemical reduction, noble metals

## Abstract

The process of noble metal nanoparticle synthesis is complex and consists of at least two steps: slow nucleation and fast autocatalytic growth. The kinetics of these two processes depends on the reductant “power” and the addition of stabilizers, as well as other factors (e.g., temperature, pH, ionic strength). Knowing these parameters, it is possible to synthesize materials with appropriate physicochemical properties, which can be simply adjusted by the type of the used metal, particle morphology and surface property. This, in turn, affects the possibility of their applications in various areas of life, including medicine, catalysis, engineering, fuel cells, etc. However, in some cases, the standard route, i.e., the chemical reduction of a metal precursor carried out in the batch reactor, is not sufficient due to problems with temperature control, properties of reagents, unstable or dangerous intermediates and products, etc. Therefore, in this review, we focused on an alternative approach to their chemical synthesis provided by microreactor systems. The use of microreactors for the synthesis of noble metal nanomaterials (e.g., Ag, Au, Pt, Pd), obtained by chemical reduction, is analyzed, taking into account investigations carried out in recent years. A particular emphasis is placed on the processes in which the use of microreactors removed the limitations associated with synthesis in a batch reactor. Moreover, the opportunities and challenges related to the synthesis of noble nanomaterials in the microreactor system are underlined. This review discusses the advantages as well as the problems of nanoparticle synthesis in microreactors.

## 1. Introduction

**Figure 1:**
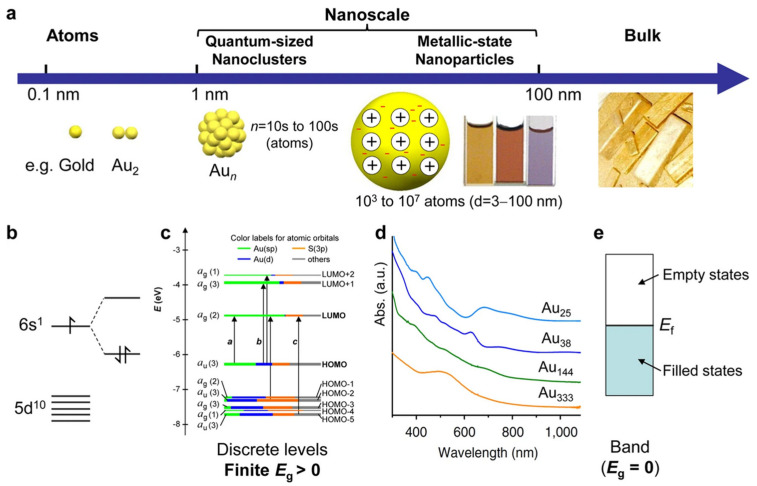
The evolution from atomic building blocks to the nanoscale, and then to bulk metals. (**a**) The nanoscale (1–100 nm); (**b**) atomic and diatomic electronic states; (**c**) molecule-like electronic structure in quantum-sized nanoclusters (where HOMO = highest occupied molecular orbital, LUMO = lowest unoccupied molecular orbital, Eg = HOMO-LUMO gap); (**d**) evolution from discrete electronic excitation to collective electron excitation (plasmon) in optical absorption spectra with increasing size of nanoclusters; (**e**) continuous band electronic structure of metallic-state nanoparticles and bulk metals (where Ef = Fermi level/energy). Reprinted licensed under Creative Commons CC BY [1].

Noble metal nanoparticles, like silver, gold, palladium and platinum, have unique physicochemical properties (optical, electrochemical, catalytic, etc.) which are quite different from their bulk counterparts. Bulk Ag, Au, Pd, Pt are shiny, malleable, tractable and highly conducting. The physical and chemical properties of these metals do not change so much with decreasing dimensions down to the nanoscale. The size of 100 nm is taken as the boundary between the typical properties of materials on a larger scale and the unusual properties offered by the nanomaterials. Moreover, nanoscales can be split into two size regions (Figure 1). The first one, known as a quantum size, contains 1–3 nm particles (tens to hundreds of atoms). Quantum-sized nanoclusters behave as molecules and have a molecule-like electronic structure (Figure 1c). The second one consists of a regular metallic-state nanoparticle with a size in the range of 3–100 nm [1].

The selected properties of Ag, Au, Pd and Pt at the bulk and at the nanoscale are summarized in Table 1.

In Table 1, the most interesting properties are selected. The first one is the difference in melting temperature, which at nanoscale depends on size [17] and even particle shape [16]. The differences in the temperature change all the existing phase diagrams developed for bulk materials. Of note, the existing phase diagram for the nanoscale is mostly based on density functional theory, Monte Carlo and molecular dynamics simulations. This is due to difficulties in preparing an appropriate amount of material for thermodynamic tests.

However, phase diagram information is crucial for these metals’ applications in catalysis. Binary systems, e.g., Ag-Au [18], and more complex ternary phase diagrams at the nanoscale are different, and this fact should be taken into account in the process of a catalyst design.

**Figure 2:**
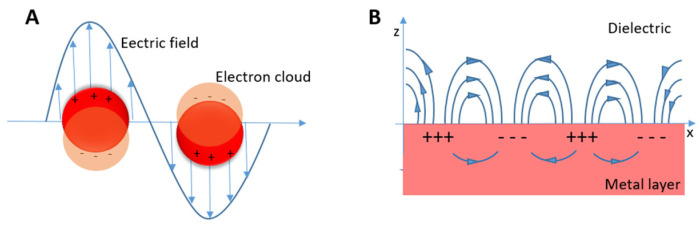
Localized surface plasmon resonance (**A**) vs. surface plasmon polariton (or propagating plasmon) in thin metal layer (**B**).

The second important difference is the occurrence of localized surface plasmon resonance (LSPR) [19]. At this point, it is worth comparing LSPR with surface plasmon polariton (SPP) (Figure 2). LSPR is characteristic for solid nano-objects dispersed in a liquid phase [20,21] and is one of the most interesting physical properties of nanoparticles, whereas SPP is dedicated to the solid nanolayer [22].

Localized surface plasmon resonance occurs when the frequency of the incident photon matches the collective oscillations of conduction electrons in metallic nanoparticles [22]. LSPR gives insights into particle morphology (size, shape), especially for silver and gold nanoparticles which have a wide range of colloidal dispersion colors (see Table 1). The particles’ morphology and their evolution under the influence of a stimulus are crucial for further nanoparticle applications. For example, the interaction between metallic particles and analytes leads to the application of LSPR in sensing [23,24], and it can be detected using spectrophotometry.

Thus, the last decade was devoted to the synthesis of nanoparticles, their characterization and applications. A Scopus analysis of Topic Cluster TC. 700: Nanoparticles; Metal Nanoparticles; Nanostructures, showed that in the years 2013–2022, more than 33 thousand papers were published with a citation level of about 900 thousand. The Considered Topic Cluster is located at the 92.2 percentage of top prominence.

Contrary to this topic, microreactors are not so popular. This is due to the necessity of having appropriate equipment, which of course involves costs. However, for more “specific” reaction conditions, it is better to apply microreactor systems. The application of microreactor systems removes most limitations typical for batch reactors (applied in the chemical reduction method), like a problem with temperature control, evaporation, dangerous and toxic intermediates, control of unstable products, etc. Thus, the use of microreactor systems for noble nanoparticle synthesis is sometimes only one possibility to make the process come true.

The main aim of this work is to overview the most recent applications of microreactors in the process of noble metal nanoparticle synthesis, as well as functionalized materials containing these particles. This review focuses on four main areas. The first one relates to noble metal nanoparticles (Section 2). This part contains description of the methods of synthesis; properties; and description of the most useful model for the mechanism of nanoparticle formation, highlighting the underestimated side of process kinetics and its complexity. The second one contains the state of the art in the field of microreactors (Section 3), methods of fabrication; the application potential; and advantages and disadvantages of a process carried out in the microreactor systems comparing to a batch reactor. The next part of this review (Section 4) contains a brief description of noble metal nanoparticles synthesized using microreactor systems, underlying the most important findings. Finally, the last part (Section 5) contains suggestions/perspectives for the future applications of microreactors for the synthesis of nanomaterials.

## 2. Noble Metal Nanoparticle Synthesis

**Figure 3:**
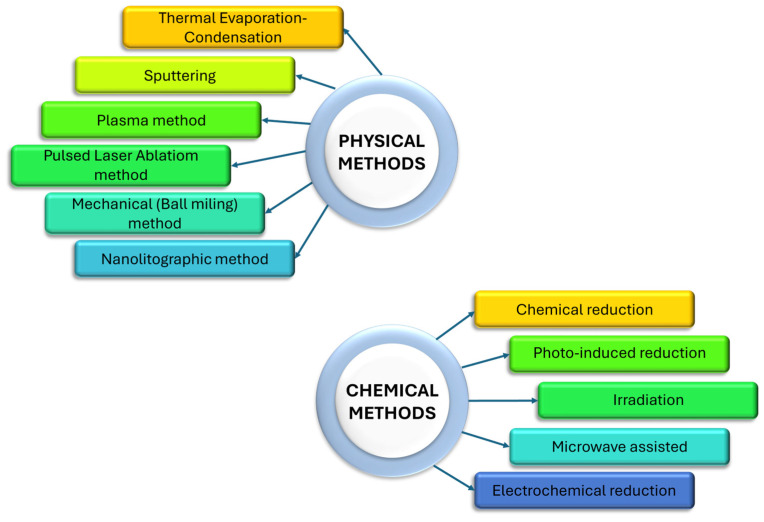
Nanomaterial synthesis methods.

The process of nanoparticle synthesis can be obtained using different methods. Their variety allows for obtaining nanomaterials with desirable morphology and properties. In general, the processes of noble metal nanoparticle synthesis are divided into chemical and physical routes (see Figure 3), which will now be shortly described.

### 2.1. Chemical Methods

**Figure 4:**
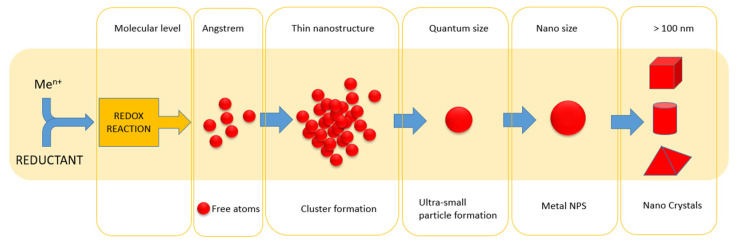
The process of nanoparticle synthesis using chemical reduction method. Adopted from [26] under Creative Commons license CC BY.

Chemical methods are the most commonly used techniques allowing one to obtain particles with nanometric size and different shapes (see Table 2). This method contains a chemical reduction, a photo-induced reduction, an irradiation, a microwave-assisted synthesis and an electrochemical synthesis [25]. Among them, the most common technique is the chemical reduction method (see Figure 4).

The chemical reduction method is cheap, fast and does not require special equipment (the synthesis is carried out mostly in the batch reactor made of glass). In the process of nanoparticle synthesis, solutions containing metal ions are mixed with a reducing agent. After that, the reduction reaction of the metal precursor takes place, whereas the reductant is oxidized. In a simple case, the reduction process can be one step (e.g., Ag(I) is reduced to Ag(0)), or two steps (e.g., Au(III) is reduced to Au(I), then to Au(0)). These steps can be more complex if reaction conditions (pH, T) allow for the occurrence of another process, e.g., hydrolysis. The process of Au(III) hydrolysis has a strong influence on metal precursor form(s), and thus on the rate of the process, as well as the final particles’ morphology. Mostly, this process is usually neglected, and more attention is paid to the environmental conditions that favor the reduction process. However, the hydrolysis process is strongly related to the pH of the environment and is mainly responsible for the rate of reduction. The second neglected aspect is the presence of gases dissolved in aqueous solutions. The influence of oxygen dissolved in a water base solution was studied for Pt(IV) ion reduction. This metal was reduced by sodium borohydride [27] and ascorbic acid [28]. In both cases, the presence of oxygen changed the reaction rate and the mechanism. However, a more complex process was observed in the case of ascorbic acid, which reacts in parallel with oxygen and the Pt(IV) complex [28]. The choice of reductant also plays an important part in the process of metal nanoparticle formation. These agents are split into two groups: mild and strong. The example of mild reducing properties is given in the case of ascorbic acid and sodium citrate (Turkevich procedure). These agents donate electrons which are accepted by an oxidant. Besides the redox reaction, the reducing agents and/or their oxidized product are a source of electrical charge. This charge is desirable in the synthesis of colloidal particles and is responsible for maintaining their stability, called electrostatic stabilization. Depending on the reaction conditions, it is possible to obtain different metal particle morphologies. The best-known example of a strong reducing agent is sodium borohydride and sodium hydrazine [29,30,31]. The use of sodium borohydride leads to ultra-fast metal reduction within a few ms [32], and this agent is often used when seeds are needed (ultra-small particles), e.g., in the process of Ag, Ag-Au [33,34] nanorod synthesis. In many cases, sodium borohydride is an efficient reducing agent, but must be used as a fresh and “ice-cold” reagent (in a cold solution to protect its decomposition). In addition, usually in the process of particle formation, a capping agent must be added to protect particles from their further growth. In the considered chemical reduction method, the presence of oxygen dissolved in the solution is neglected to simplify the initial reacting system (solution) to a liquid phase. Thus, the process of metal ion reduction takes place in a homogeneous system. The last step in this reduction process leads to the appearance of free atoms. This step also changes the system from a homogeneous (only liquid) to heterogeneous phase, and the nucleation process takes place at the liquid–solid interface. The nucleation process is very complex, and it consists of the structural transformation from atoms to nanoparticles (atoms => thin cluster => cluster with core = > ultra-small particles => nanoparticles (diameter (D) > 2 nm) [26]. The inner processes of nucleation tend to be physical in nature; atoms connect to each other to form metastable clusters. These clusters might grow to form larger objects (10–30 nm) or they might break down. This decay is actually characterized by the breaking of smaller clusters and the incorporation of atoms into the structure of a larger cluster by their attraction forces (large objects attract smaller ones).

A special case among chemical reduction methods is the polyol method. Polyol method synthesis is dedicated to the preparation of easily reducible cations, such as Ag(I), Cu(II) and Ni(II) [35,36]. This synthesis involves a selection of a metalorganic compound. Due to their nature, metalorganic compounds allow control of the process of particle formation by mild reaction conditions. Additionally, by using them, the contamination from other ions or halide is highly limited. Although organometallic complexes have many advantages, they are difficult to obtain, have a high price, and generally tend to decompose [37]. In the polyol method, the reacting medium is used as a reducing agent and as a solvent in which the metal salts are dissolved. For example, poly(vinylpyrrolidone) (PVP) has been often used to passivate the surface of silver nanoparticles to protect them from being sintered. This synthesis can proceed at room temperature, though higher temperatures are usually used for higher reaction rates [38,39]. The formation of nanoparticles can be split into at least two steps (as was previously described). The first one relates to the reduction of metal ions and the second one to their homogeneous nucleation. The example of Ag nanoparticles shows that the first stage starts by reducing Ag^+^ to Ag(0). Next the reduced metallic form of Ag coagulates into larger groups, forming clusters. With time, the growing clusters evolve into nanoparticles. To prevent the overgrowth of nanoparticles, it is crucial to select an appropriate type of stabilization for the synthesized nanocolloids. The overview of different factors influencing the process of noble metal nanoparticle synthesis is gathered in Table 2.

**Table 2:** Selected critical parameters of nanoparticle synthesis with different reducing agents.

Type of Metal	Precursor	Reductant	Conditions	Shape [40]	Size, nm	Ref.
Ag	AgNO_3_	sodium borohydride	pH: 2–10; solvent: water	non-uniform	8.25	[41]
			solvent: water	nanoprisms	3.8	[42]
		ethylene glycol	solvent: ethylene glycol; 148 °C	truncated cube/ tetrahedron	60–80	[43]
		oleic acid	solvent: 1-octanol; 180 °C	non-uniform	2–22	[44]
		dihydrated trisodium citrate	solvent: water; 60 °C	quasi-spherical	20–50	[45]
		*Daucus Carota* (carrot extracts served as reducing and closing agents)	pH: 5.5–8; solvent: water; 24 °C	triangular, cubic, polygons or rods	10–35	[46]
		Fungus *Aspergillus niger* (after growing the fungus in nutrient medium (potato dextrose broth), the biomass was filtered. The filtrate contains extracellular bioactive compounds that act as reducing and stabilizing agents)	solvent: water; 25 °C	spherical	1–20	[47]
		carboxymethylated chitosan (CMCTS)	pH: 0.7, 2.2, 9.6, 12.4; solvent: water	-	2–10	[48]
		hydrazine hydrate, ascorbic acid	solvent: formaline; 22–25 °C	spherical	20<	[49]
		UV irradiation	solvent: water; 25 °C	cubes, rods and spheres	11.7- 77.8	[50]
		sodium borohydride, ascorbic acid and sodium citrate	solvent: water; 5, 20, 30, 40, 50, 85 °C	non-uniform	8<	[51]
		extract from the fruits of *Santalum album* (crushed fruits were mixed with ethanol, exposed to microwave irradiation, then combined with an aqueous AgNO_3_)	solvent: water; 25 °C	-	24–40	[52]
		ethylene glycol monoalkyl ether	solvent: ethylene glycol monoalkyl ether; 120 °C	prisms, plates	45–129	[53]
		ethylene glycol	poly(vinylpyrrolidone); 70–90 °C	nearly spherical	97–113	[54]
		*Bacillus Strain CS 11* (bacterial strain (CS 11), obtained from soil polluted with heavy metals, was identified as a strain of Bacillus sp. The bacterial strain was treated with 1 mM AgNO_3_, resulting in the formation of AgNPs)	0.8% NaCl; 25 °C	spherical	42–94	[55]
		leaf extracts from *Eucalyptus macrocarpa* (the cleaned leaves were cut into small strips and immersed in 100 mL of water; the mixture was homogenized. The solution was filtered to obtain a clear leaf extract)	solvent: water; 24 °C	cubic	10–50	[56]
		*Lactobacillus* strains (growing in MRS medium, cells were collected by centrifugation. Isolation of capsular exopolysaccharides by treatment of bacterial cells with phenol, precipitation with ethanol and dialysis. Acted as reducing agents)	solvent: water; 37 °C	spherical	10–40	[57]
		bacteria *Shewaneila oneidensis MR-1* (the bacterium was grown on Luria–Bertani agar at 30 °C. Single colony was inoculated for 24 h with shaking. Bacteria were collected by centrifugation and washed with distilled water and 3–5 g of wet biomass was incubated in 1 mM AgNO_3_ solution)	bacterial biomass; 30 °C	spherical	4–11	[58]
		N,N-dimethylformamide	solvent: N,N-dimethylformamide; 156 °C	nanoprisms	30–200	[59]
	Silver rods 2 mm	electrical arc discharge	solvent: water	spheres, triangles and polygons	10–120	[60]
Au	HAuCl_4_	sodium citrate	pH: 2.45; solvent: water;	shell	1.8, 15,	[61]
			pH: 7; solvent: water; 25 °C	spherical	18.8, 28.2	[62]
		sodium borohydride	solvent: methanol; 25 °C	spherical	1.3	[63]
		black phosphorus	solvent: water; 25 °C	spherical	28	[64]
		glutathione/cysteamine	pH: 4.8–8; solvent: water; 37 °C	-	2.7, 3.1	[65]
		tannic acid/citrate mixtures	pH: 8; K_2_CO_3_	quasi-spherical	3.5–10	[66]
		ascorbic acid	solvent: water; 25 °C	spherical, star shape	about 20	[67]
		tetrakis (hydroxymethyl) phosphonium chloride/citrate mixtures	solvent: water; 25 °C	nanoshell	1–20	[68]
	AuCl_3_	trisodium citrate dihydrate	pH: 3, 4, 5, 7; solvent: water; 25 °C	non-uniform	11.7	[69]
	Au(I) Thiolate Precursor	sodium borohydride	pH: 5.5–8; solvent: water; 25 °C	core−shell	2–6	[70]
	H_3_AuCl_4_O	CTAB/ DPPC liposomes	pH: 4–11, 13; solvent: water	nanowire	50–70, 25	[65]
Pd	PdCl_2_	hydrochloric acid	pH: 4, 6, 9, 11; solvent: water; 25–85 °C.	non-uniform	3.14	[71]
		guar gum (1 g of guar gum powder was dissolved in 50 mL of ethanol (EtOH) at room temperature, then 10 mg of PdCl_2_ was added)	solvent: ethanol; T: 25 °C	cubic	70–80	[72]
		perchloric acid	perchloric acid	rods	100	[73]
		anodization	CuCl_2_ · 2H_2_O, 0.1 M HCl	spring	60, 70, 250	[74]
		root extract of *Salvadora persica L. (Miswak)* (fresh roots were cut into small pieces and soaked in deionized water, boiled for 4 h and filtered, and the extract dried under vacuum. Reduced with PdCl_2_ at 90 °C)	solvent: water; 90 °C.	spherical	2.2–15	[75]
	Pd(NH_3_)_4_Cl_2_	anodization	pH: 8.5; Na_2_EDTA; T: 25 °C.	wire	80	[76]
	Na_2_PdCl_4_	ethylene glycol	solvent: ethylene glycol; 85 °C.	triangular plates	28	[77]
			solvent: ethylene glycol; 160 °C.	spherical	6, 24.38	[78]
		l-ascorbic acid, citric acid	solvent: water; 100 °C.	truncated octahedral	9.1	[40]
		formic acid	solvent: water; 60 °C	cubic	14–37	[79]
Pt	H_2_PtCl_6_	sodium borohydride	pH: −1–15; solvent: ethanol; 90 °C	spherical	1.76–3.26	[80]
		polyethylenimine	pH: 1–12; solvent: water; 60 °C		10	[81]
		Ajwa and Barni date extracts (extracts were prepared by heating them in the dark at 100 °C, filtered and mixed with a solution of H_2_PtCl_6_)	pH: 1.5–8.5; solvent: water; 90 °C		2.3–13.8	[82]
		roots of *Ononidis radix* (extract was prepared by adding 3 g of O. radix powder to 100 mL of distilled water, boiled and stirred for 1 h at T = 85 °C)	solvent: water; 85 °C	spherical and hexagonal	4	[83]
		leaves of *Doipyros Kaki* (leaves were collected, washed thoroughly, dried for two days at room temperature and finely cut. Then, 5 g of these dried leaves was boiled in 100 mL in distilled water for 5 min and the solution was decanted and stored at 4 °C)	solvent: water; 25–90 °C	spheres and plates	2–12	[84]
		tea polyphenol	solvent: tea polyphenol; 25 °C	flower	30–60	[85]
	K_2_PtCl_4_	formic acid	solvent: water	dendrites	20–50	[86]
		sodium borohydride	solvent: water; 50 °C	cubic	11.55	[87]
		ascorbic acid	solvent: ethylene glycol; 50 °C	spherical	1.5–4.5	[88]
	PtCl_4_	sodium borohydride	solvent: ethanol; 25 °C	spherical	2.2–3.3	[89]

### 2.2. Physical Methods

An alternative method to chemical synthesis is synthesis via a physical route. Two main methods could be given, namely bottom-up and bottom-down. Both of them are based on the application of mechanical pressure, high energy radiation, thermal energy or electrical energy. These operations are required to cause material abrasion, melting, evaporation or condensation to create NPs. Many of the listed above methods have beneficial features like less time needed (fast rate of the processes), no toxic chemicals, high quality of obtained NPs and uniform dimensions of NPs in production process (size and shape of NPs). They also have many disadvantages. The main drawbacks are a worse optimization of the process, high cost of production related to process requirements (energy consumption, high temperature and pressure), difficulty in maintaining thermal stability and high amount of post-production waste. All these features delineate the limitations of physical methods [25,90,91].

### 2.3. Mechanism of Nanoparticle Formation

Many attempts have been made to describe the mechanism and the process of nanoparticle formation using mathematical equations. Recently, Whitehead et al. [92,93] collected all the most important works related to this issue, underlying the progress of the existing models and their adoption to different systems, e.g., sulfur sol, silver nanoparticles, metal oxide, semiconductors or quantum dots. The aim of these models is to better understand the nature of nucleation and growth processes of different types of particles. Consequently, through discovering the mechanism, one can find the answer to which kinetic factor allows the control of particle size and dispersity.

#### 2.3.1. Watzky–Finke Model

The process of nanoparticle synthesis according to the W–F mechanism is based on two processes. The first one is a slow nucleation process defined as being low-level and continuous. The second one includes a high-rate stage related to autocatalytic growth at the cluster surface. The classical W–F model was dedicated to iridium particle formation in a two-step process: Equations (1) and (2).

(1) $$A\overset{k_{n}}{\to }B$$

(2) $$A+B\overset{k_{g}}{\to }2B$$

where A—metal precursor, B—cluster active surface, k_n_—constant rate of cluster nucleation and k_g_—growth rate constant.

The comparison of this model to the classical LaMer mechanism indicates that autocatalytic surface growth is not limited by diffusion. For this reason, the two-stage W–F model can be extended for multi-stage reduction and further growth, with possible aggregation leading to large solid-phase formations (above the nanoscale) [94]. Due to the difficulty associated with determining the kinetic equation for a multi-stage reaction process, only the first two stages are commonly used.

It is worth mentioning that the reduction step is often neglected, especially for rapid reactions [95,96]. However, the W–F model provides a mechanism to explain nucleation and growth. Moreover, it does not consider the role of ligands in the reaction process. Though this aspect at first has been neglected, a new approach for adapting the W–F model has been developed. In the literature, the crucial role of the ligands and their relation between nucleation and growth kinetics, such as slowing down the particle growth by blocking surface binding sites or slowing down the reduction and growth of nanoparticles [97,98], can be found. This relation can be expressed by the following reactions:

(3) $$A\overset{k_{1-nuc}}{\to }B$$

(4) $$A+B\overset{k_{2-growth}}{\to }2B$$

(5) $$A+L\overset{\overset{k_{3-f},K_{5-eq}}{\to }}{\leftarrow }AL$$

(6) $$B+L\overset{\overset{k_{4-f},K_{6-eq}}{\to }}{\leftarrow }BL$$

where k_1-nuc_ is the reaction rate constant for the combined pseudo-elementary reduction–nucleation step in the reduction–nucleation process (3); k_2-growth_ is the reaction rate constant for the autocatalytic surface growth (4); k_3-f_ is the forward reaction rate constant for the precursor–ligand association step; K_5-eq_ is the equilibrium constant for the reaction of the ligand binding to the precursor; k_4-f_ is the forward reaction rate constant for the particle–ligand association step; K_6-eq_ is the equilibrium constant for the ligand binding to the particles’ surface; A represents the precursor; L is the free ligand (TOP) in a solution; and AL is the complex which is coordinated with the other ligands; B is palladium surface atom; BL is the ligand-capped metal surface atom [99].

Working with this model, it is important to remember the difference between coalescence and orientated attachment. The orientation attachment is dictated by the preference for the crystal lattice at the grain boundary, while, for the coalescence, the attachment is random [100].

In the case of noble metal nanoparticle synthesis, the most useful model is that of Watzke and Finke. This model or its modified form was successfully adopted to the process of nanosilver [101,102], gold [103], palladium [104] and platinum [32] particle formation.

At this point, it is worth mentioning that there is another method of material synthesis based on seeds. It is known as the seed-mediated [105] growth method and involves the creation of small nuclei that act as the nucleation center for the particles. The addition of a surfactant, e.g., cetyltrimethylammonium bromide (CTAB), blocks selected growth directions, causing the particle to grow in a controlled manner in only one direction. This method allows for nanorods synthesis, for example. Recently, techniques based on iodine ions [106] or bromide-free techniques [107] have been developed, which eliminate the need to introduce CTAB. The proposition of the mechanism of seed-mediated particle growth is described in the literature [108], but is not supported by kinetic data necessary to design the process in flow (see Section 2.3.2).

#### 2.3.2. Kinetics of Reduction, Nucleation and Growth—Why Are They Important?

The process of noble metal nanoparticle formation is strictly related to the synthesis procedure in which kinetics and thermodynamics play key roles. Thermodynamics gives an answer to the question of whether the process of particle formation may occur. In turn, kinetics tells us how fast it will be. Taking into account one of the most commonly used thermodynamic function like Gibbs energy, which is expressed as

(7) ∆G=∆H−T∆S

where ∆G—Gibbs energy change, J·mol^−1^; ∆H—enthalpy change, J·mol^−1^; *T*—temperature, K; ∆S—entropy change, J·mol^−1^K^−1^, one can divide each process into possible (∆G<0) or impossible (∆G>0) categories. In turn, kinetic studies give information about the rate of the process. Moreover, the results obtained from the kinetic studies (like the shape of the kinetic curve and rate constants) suggest the mechanism of the process.

To properly proceed with the design of nanomaterials (especially nanoparticles with a defined morphology) kinetic parameters are required. Knowing the observed rate constants, it is possible to determine the time needed for the reaction to be completed. Additionally, there exists an option to give information on at what stage at what given moment the reaction proceeds. This, in turn, is very important for process designing, especially when it is carried out in the flow, e.g., in the microreactor. Here, a very important parameter is the flow rate, which can be set by the operator, and it is connected with time by the following equation:

(8) $$F_{R}=\frac{V}{t}$$

where $F_{R}$—flow rate, mL/min; V—reactor’s volume, mL; t—time, min.

The residence time of the reactants in the microreactor can be determined from the kinetic equations of individual stages during the process of the nanoparticle formation.

## 3. Microreactors—Present State-of-the-Art Technology

In recent years, significant advances in science and technology have contributed to the progress in the chemical engineering industry, particularly in the realm of chemical reactors. Currently, much attention is being paid to microreactors and microreactor-based devices, which offer promising opportunities for highly efficient chemical synthesis methods [109]. The microreactor is signed as a miniaturized chemical reactor, having internal channels with dimensions ranging from sub-micrometer to sub-millimeter scale. The microreactor channels are fabricated through precision engineering or microtechnology on flat surfaces and resemble integrated circuits [110,111]. Reactions in microfluidics can be carried out using microchannels on a chip and in tubes with microchannels made of stainless steel, PTFE poly(1,1,2,2-tetrafluoroethylene) or other materials [112]. Nevertheless, current microreactor technology includes the integration of additional microdevices and control mechanisms aimed at the precise regulation of reaction conditions, encompassing parameters like the temperature, pressure and residence time of the reaction mixture within the device [109,110]. The date of the design of the first microreactor is not strictly defined, because a capillary with microchannels can be also considered as a microreactor. However, several facts in history are closely related to the current perception of microreactor technology. The first noteworthy event is the publication on fluid mechanics in 1883 in the *Philosophical Transactions of the Royal Society*. This paper [113] is related to today’s well-known laminar and turbulent flow used in current engineering, including microreactor technology. The establishment of the American Microchemical Society in 1934 also played a significant role in the evolution of microreactors. A German patent in 1984 marked an early description of microreactor technology, with the first document concerning its industrial application appearing in 1993. Interest in microreactor technology increased after the publication of the concept of chemical integration using the microreactor in an “Integrated Chemical System”. Presently, technology is rapidly developing mainly in the scientific community, offering more complex microreactor circuits and expanding the applicability of such devices. Developments in the field of microreactors make it possible to use them for liquid-phase reactions, multiphase reactions, catalysts and nanomaterials [110]. This review will present the developments in the field of microreactors over the past few years, specifically covering the materials and processes used to manufacture microreactors, as well as applications in material synthesis [114].

### 3.1. Fabrication of the Microreactor

The choice of fabrication techniques and materials for microreactor production is guided by many factors. Among them, the most important are the following: chemical compatibility with reagents, temperature requirements and desired functionalities of the system containing the microreactor. The modern microreactor-manufacturing techniques enable the production of devices distinguished by their compactness and high performance. This technology provides a diverse range of fabrication methods, which depend on the substrate material chosen and the complexity of the system being developed. Techniques include lithography, etching, micromachining and 3D printing.

***Lithography (LIGA)***. The LIGA fabrication technique is one of the most popular methods used for producing microreactors due to the possibility of using different materials, such as polymers, metals and plastics. The technique, referring to the German acronym LIGA (*Lithographie, Galvanik and Abformung)*, comprises three fundamental process steps. Initially, the system’s pattern is transferred onto a photoresist, commonly a photosensitive epoxy resin. Next, unwanted materials are removed and the substrate surface is formed via electrocoating, involving electrolytic coating. In the final stage, the metallic component is fashioned, and the photoresist is eliminated. Depending on the light source for photoresist removal, a distinction can be made between photolithography, whose light source is visible light, ultraviolet or extreme ultraviolet light, and X-ray lithography. An alternative lithography method, characterized by a more cost-effective fabrication process compared to the aforementioned techniques, is soft lithography. It relies on creating channels within the size range of 30 nm to 100 μL using cast stamps composed of elastomeric polymers [80,115].

***Micromachining***. Micromachining includes the manufacture of microfluidic devices and other types of microreactors. Generally, the term used for these technologies is micro-electro-mechanical systems (MEMSs). Micromachining involves wet or dry etching, laser ablation or drilling to create microstructures directly on materials such as glass, silicon or polymers. The surface quality obtained from these processes is very different and depends on the material used and the processing parameters. Depending on the material, precision machining is carried out by spark erosion, laser machining and precision machining. Micromachining enables the creation of high-aspect-ratio components and is often used in specialized microreactor designs. This technology is characterized by its lower cost, as compared to other techniques used for microreactors production [116].

***Etching***. Etching is a method of microreactor manufacturing that involves the selective removal of a material from the substrate to create microchannels and reaction chambers with precise geometries. The technology offers two options for conducting the process: wet chemical etching (where the material is etched in the liquid phase using 10% buffered hydrofluoric acid) and dry etching, which involves the elimination of a material transformed into a gas. The dry etching process is more costly and has a low etching rate. Etching allows for the creation of intricate microchannel patterns with precise dimensions and shapes. The technique can be applied to various materials, including silicon, glass and polymers, offering flexibility in microreactor design and application [109].

***Three-dimensional printing***. Additive manufacturing, specifically 3D printing, is gaining prominence in microreactor fabrication. This technique allows for the creation of intricate 3D microreactor architecture with precise control in a single technological step. The advantage of 3D printing results from the fact that the microreactor can be designed for a specific process and the design can be quickly modified using computer-aided design tools (e.g., CAD). A diverse range of materials can be constructed with polymers, ceramics and metals, which can be used for the 3D printing of microreactors [117].

Microreactors can be constructed with a wide range of materials, including glass, silicon, ceramics, polymers and metals (e.g., steel, aluminum, copper). The selection of materials depends on several factors, including operating conditions (pressure and temperature), substrate properties (phase, pH, reactivity, etc.) and manufacturing cost [118].

Silicon stands as the most commonly used material due to its availability and cost-effectiveness. Moreover, silicon-based microreactors allow excellent laminar flow, particularly advantageous for interfacial reactions. However, a disadvantage is their opacity, which makes them incompatible with optical detection systems in the visible range, making it difficult to observe reaction in real-time. In contrast, quartz or glass microreactors offer excellent optical properties. Quartz and glass also have additional advantages, such as high temperature and chemical resistance. The glass microreactors can withstand pressures (up to 4107 Pa) and exhibit good resistance to various solvents, making them suitable for organic syntheses and facilitating electro-osmotic flow. Ceramic microreactors are well suited for high-temperature reactions and provide high corrosion resistance. However, their brittleness and porosity may limit some applications. Metals and their alloys, including iron, stainless steel, aluminum and copper, are also good choices for microreactor design. They offer efficient heat and mass transfer properties, along with excellent mechanical strength. The diverse range of metals and alloys allows for the selection of suitable materials based on specific reaction conditions. In recent years, there has been increased attention on certain polymeric materials suitable for microreactors, such as polydimethylsiloxanes and their modified versions, thermoplastic polymers (e.g., polystyrene, polyvinyl chloride, polycarbonate) and olefin-based polymers. Polydimethylsiloxane, in particular, exhibits favorable chemical, physical and optical properties, including chemical inertness, water resistance, gas permeability and a low elastic modulus [115,116]. However, its limitation is its tendency to swell in the presence of organic solvents. The advantages and disadvantages of typical microreactor materials are gathered in Table 3.

Promising materials include cermets, combining the advantageous properties of metals and ceramics, as well as hybrid materials that blend multiple material benefits. The strategic combination of the best attributes from existing materials holds significant potential for designing highly efficient and miniaturized reaction systems [115].

Developments in manufacturing technologies continue to expand the capabilities of microreactors, enabling the development of highly efficient and customized microreactors for various industries and research concepts. Advances in microreactor-based systems can contribute to a variety of fields, including chemical synthesis, biotechnology and material science [109].

### 3.2. Microreactor vs. Batch Reactor

The microreactor exhibits numerous advantages over traditional batch reactors, stemming from its design and miniaturized scale. Microreactors can operate in a continuous flow mode, enabling a constant and steady supply of reactants and continuous product output. This continuous flow setup intensifies the conducted chemical process and increases productivity. The processes carried out in microreactors allow for precise control over reaction parameters. Reactions proceed in microreactors characterized by a high surface-to-volume ratio, which promotes efficient heat and mass transfer during the process [119]. The small dimensions of microchannels facilitate rapid heat exchange between the reactants, leading to better temperature control and reduced temperature gradients. Increased heat transfer increases process efficiency as compared to conventional batch reactors [120]. The design and construction of microreactors significantly improve reactant mixing. The laminar flow within microchannels ensures a uniform distribution of reactants, reducing concentration gradients. Better mixing leads to increased reaction efficiency and higher selectivity of the final product [121,122]. Microreactors require smaller quantities of reagents. This makes the chemical processes safer for operators and also decreases the generation of hazardous waste. The minimized reagent and solvent volumes promote more sustainable and environmentally friendly chemical processing. The integration of microreactors with analytical tools enables real-time monitoring of reaction progress. This capability allows for precise process control and optimization, enhancing the overall efficiency of chemical synthesis [111,123,124]. However, microreactor technology also has some drawbacks. These include the formation of undissolved reaction products, which can accumulate and settle in the microchannels of the reactor. Additionally, the generation of gaseous components during the reaction process can contribute to the destabilization of the continuous flow of reagents [119]. These factors can potentially hinder the overall performance and operation of microreactors in some chemical processes.

**Figure 5:**
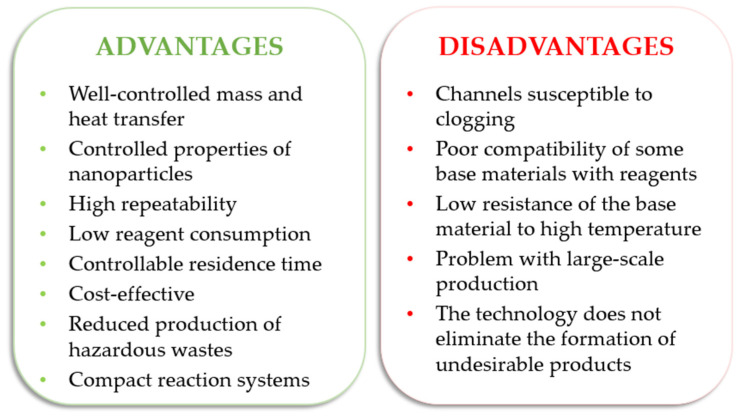
Advantages and disadvantages of microreactor systems [125].

The main advantages and disadvantages of microreactors are shown in Figure 5. Ongoing research is aimed at overcoming challenges and optimizing microreactor performance in various applications.

### 3.3. Types of Microreactors

Microreactors offer numerous advantages over traditional batch processes, particularly in terms of their ability to perform complex reactions with excellent control over kinetics and product properties by optimizing synthesis conditions. They find extensive applications in various fields, including organic compound synthesis [126,127,128], medication and biomedicine [129,130]. In recent years, microreactor technologies have gained importance in the synthesis of nanoparticles, including inorganic metal nanoparticles, semiconductor nanomaterials and quantum dots [131,132,133]. Microreactor technology enables the precise control of flow rates and temperatures, leading to better control of growth parameters and the synthesis of nanomaterials with specific shapes and sizes [134,135]. Microreactors can be classified into two main categories: continuous-flow microreactors and segmented-flow microreactors.

#### 3.3.1. Continuous Flow Microreactors

**Figure 6:**
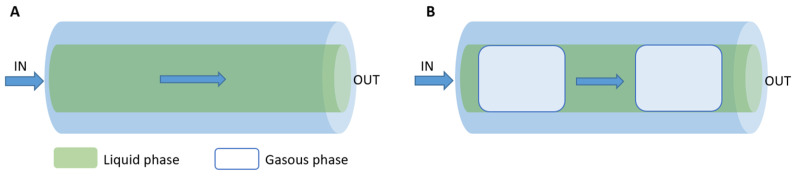
(**A**) Continuous flow microreactor (fragment of the microchannel), (**B**) Segmented flow reactor (fragment of the microchannel).

Continuous flow microreactors consist of an internally connected network of microchannels. This type of device allows for more efficient reactions and also improves the homogeneity of the final product. In continuous flow microreactors (shown in Figure 6), the reactants are continuously fed into the system, and the products are also collected continuously. The chemical composition of the reaction mixture can be changed along the reaction channel [136]. The modularity of the continuous flow microreactors allows multi-stage processes by combining different reactors [137]. Compared to segmented-flow reactors, continuous-flow microreactors have a higher capacity [138]. Microchannel architectures providing continuous flow can include capillary tubes, coaxial flow and microchannel systems on a chip [136,139]. Capillary tube microreactors feature reduced-flow cross-sectional areas at the micron level [140]. They are simple to manufacture and to operate, but may encounter problems like channel blockage and high product polydispersity. To address these challenges, microfluidic devices with coaxial double tubes are used to improve mixing efficiency. The microreactor-on-a-chip design with microchannel connections often involves simple geometries, such as T- or Y-shaped configurations, with rectangular or trapezoidal cross sections. However, more complex shapes and configurations are also possible [141].

Continuous flow microreactors have been used to synthesize materials like semiconductor nanocrystals [142,143], metal oxides [144], magnetic nanoparticles [145,146] and different types of noble metal nanoparticles (see Section 4 for details).

#### 3.3.2. Segmented Flow Microreactors

**Figure 7:**
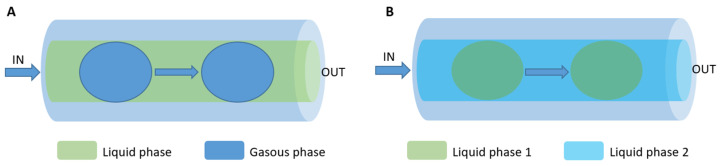
Types of segmented flow microreactor: (**A**) gas–liquid; (**B**) liquid–liquid flows.

Segmented flow microreactors are divided into segmented gas–liquid (shown in Figure 7A) and liquid–liquid (shown in Figure 7B) flows. This type of mini-reactor uses pre-flow segmentation to improve mixing [136]. The main advantage is that it provides a better size distribution of the nanoparticles. In a segmented gas–liquid flow, individual gas bubbles act as microreactors, flowing along the reaction channel at the appropriate flow rate. Multiphase gas–liquid microreactors offer the easy separation of the gas bubbles from the liquid, reducing axial dispersion. However, there is a risk of nanoparticle deposition or blockage, as they may come into contact with the channel walls. In segmented liquid–liquid flow, the precursor solution is encapsulated within the liquid droplets, avoiding interaction with the channel walls and completely preventing contamination or blockage [139].

Segmented flow microreactors are also used for the synthesis of nanocrystalline semiconductors (quantum dots). The advantage of segmented flow microreactors consists of better mixing and narrow residence time distribution, resulting in a significant improvement in reaction efficiency and size distribution [147].

Segmented flow microreactors have also been used for the controlled synthesis of metal nanoparticles.

Kulkarni et al. [148] used a continuous-flow microreactor system to synthesize gold nanoparticles (AuNPs) via interfacial synthesis. The microreactor, a silicon-Pyrex chip, consisted of two distinct zones: a mixing zone at room temperature and a reaction zone heated to 90 °C. The synthesis involved the segmented flow of an aqueous phase containing 2 mM HAuCl_4_ and 200 mM cetyltrimethylammonium bromide and an organic phase containing 5% *v*/*v* piperidine in toluene. The microchannel phases formed lumps of the aqueous phase in the continuous organic phase. The synthesis parameters were residence time, ranging from 30 s to 600 s, and temperature, ranging from 70 °C to 90 °C. At a reaction temperature of 80 °C and a residence time of 10 min, homogeneous hexagonal nanoparticles with a diagonal of 40 nm were produced. At 70 °C, smaller spherical particles of 8 nm were formed, while at 90 °C, smaller hexagonal particles with a diagonal of 30 nm were formed.

A two-phase (liquid–liquid) segmented flow system was used by Mehenni et al. [149] to synthesize silver nanotubes and silver nanospheres. Their microreactor consisted of a Teflon perfluoroalkoxy (PFA) tube, which was operated at 150 °C. The synthesis process involved an aqueous phase containing ethylene glycol with dissolved reactants including silver nitrate (AgNO_3_) at 48 mg/mL, poly(vinylpyrrolidone) at 20 mg/mL and sodium hydrosulphide (NaHS) at 3.36 × 10^−3^ mg/mL. A Fluorinert FC-70, a chemically inert liquid that facilitated segmented flow, was used in the second phase. Silver nanospheres with edge lengths ranging from 23.2 nm to 51.7 nm were synthesized by adjusting the residence time and FC-70 ratio. Shorter residence times of 20 min and higher FC-70 concentrations resulted in smaller nanotubes, while longer residence times of 120 min and lower FC-70 concentrations resulted in larger nanotubes. The silver nanospheres, with an average diameter of 31 nm, were produced by etching the nanocubes with 7.5 mM aqueous ferric nitrate solution. Monodispersed silver nanospheres were obtained by continuously adjusting the flow rate to ensure the complete transformation of the nanotubes into nanospheres.

Photochemical synthesis of platinum-group metal nanoparticles (Pd, Pt, Rh, Ir) using segmented flow was carried out by Hafermann et al. [150]. Their microreactor system consisted of PTFE tubes and transparent FEP tubes for optical analysis and photochemical initiation. Liquid segments were formed using a combined PEEK cross-linker, with aqueous-phase and water-immiscible perfluoromethyl decalin (PP9). Reagents included a 1 mM aqueous solution of noble metal salts, 2% poly(vinylpyrrolidone) and 3 mM 2-hydroxy-4′-(2-hydroxyethoxy)-2-methylpropiophenone (HMP). Flow rates ranging from 43.75 to 700 mL min^−1^ were used, allowing controlled UV-exposure times of 34 to 540 ms. The method effectively separated the mixing, nucleation and growth stages, resulting in high-quality nanoparticles with consistent sizes for a variety of metals. In synthesis obtained platinum nanoparticles with an average size of 2.5 nm, synthesized at flow rates between 87.5 and 175 mL min⁻¹, corresponding to UV-exposure times of 135 to 270 ms. Higher flow rates resulted in a broader size distribution and fewer nanoparticles due to insufficient exposure time. Palladium nanoparticles, also around 2.5 nm in size, were produced at flow rates between 87.5 and 175 mL min^−1^ with UV-exposure times of 135 to 270 ms. At a lower flow rate of 43.75 mL min^−1^ and a longer exposure time of 540 ms, the highest particle density was achieved. Rhodium nanoparticles, approximately 2.5 nm in size, were synthesized with high homogeneity at a flow rate of 175 mL min^−1^ and a UV-exposure time of 135 ms. For iridium nanoparticles, higher flow rates of 175 mL min⁻¹ and above, corresponding to UV-exposure times of 135 ms or shorter, were necessary to prevent rapid agglomeration. Under these conditions, nanoparticles of about 2.5 nm were obtained. More examples of using segmented flow for the synthesis of nanostructures are described in Section 4.2.

## 4. Noble Metal Nanoparticle Synthesis in the Microreactor Systems

**Figure 8:**
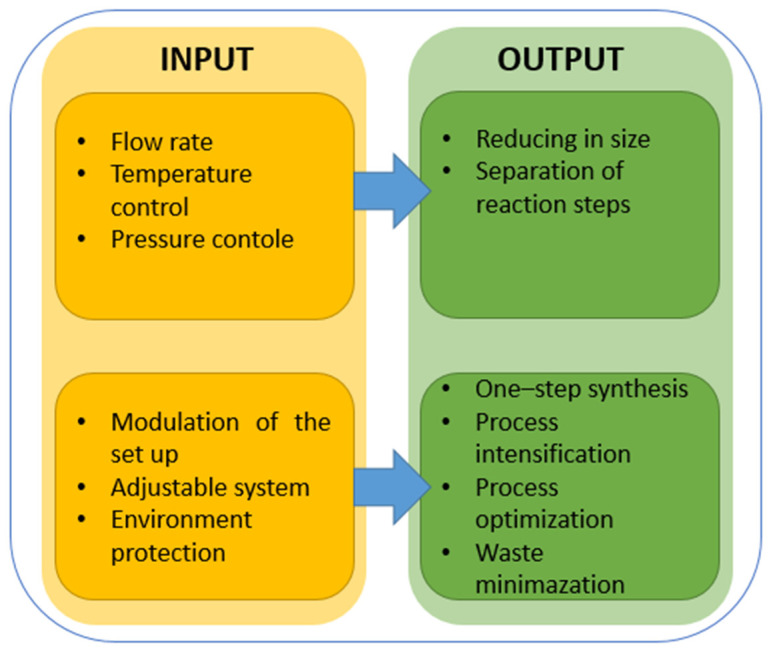
The microreactor input (set parameter control, design of the system for the individual process) and output (effect on the products and process).

For a long time, nanoparticles have been synthesized in conventional reactors. The development of microreactor systems allows for their application in the synthesis of noble metals. This technique allows the control of the size and shape of nanoparticles. Moreover, it provides better control of the process parameters. Nowadays, the synthesis of noble nanomaterials is widely described in many research papers [151]. Basically, the use of microreactors to synthesize nanomaterials has potential benefits that can be achieved. The measurable inputs and outputs of the parameters are summarized in Figure 8 and described in the next paragraphs in details (see Section 4.1, Section 4.2, Section 4.3 and Section 4.4).

In the following sections, examples will be given in which the synthesis of nanomaterials in a microreactor brought measurable benefits related to the homogeneity of the synthesized material, with the possibility of carrying out the synthesis under extreme conditions or under conditions impossible to achieve in a batch reactor. In addition, the use of microreactors as a flexible platform for controlling reaction steps will be discussed. Moreover, the possibility of using microreactors to improve the efficiency of processes, e.g., the adsorption of metal on a catalyst carrier or production catalysts, will also be described.

### 4.1. Control of Nanoparticle Morphology

**Figure 9:**
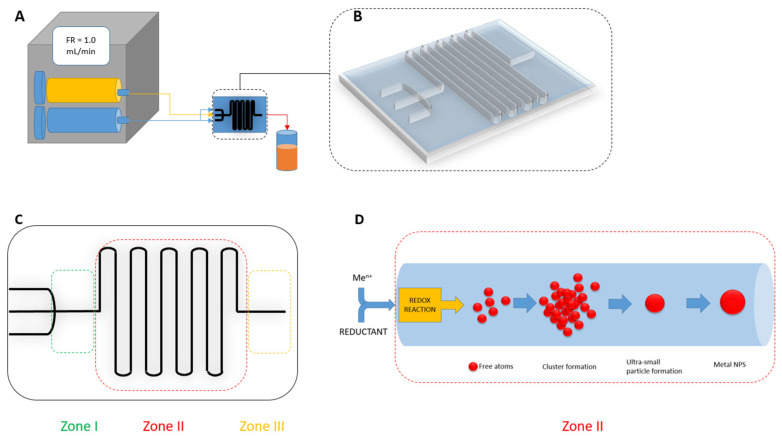
(**A**) Microreactor setup (pump, microchip, sample collector); (**B**) microreactor containing 3 inlets and 1 outlet; (**C**) cross section of the microreactor with marked zones. Zone I—reagent mixing; Zone II—reactions; Zone III—product. (**D**) Zone II contains the reduction process, slow nucleation and fast autocatalytic growth leading to stable particle formation.

Specific properties of nanoparticles result not only from their nano size in the range of 1–100 nm but also refer to their morphology and surface property (surface charge, the presence of “working” ligands, etc.). The morphology of nanoparticles is determined by their uniform size and shape. The uniform particle size can be achieved by using strong reductants like sodium borohydride. However, in such synthesis, the presence of a stabilizer is necessary due to the coalescence of the particles leading to the formation of larger particle clusters or the interaction between the particles and the oxidation products leading to the formation of aggregates. The addition of sodium citrate, ascorbic acids, etc. as electrostatic stabilizers prevents further particle growth and aggregation processes. These agents are also known as metal ion reductants, but are not as reactive as sodium borohydride. Sometimes, the process of particle growth is desirable. For example, in a seed-mediated method to obtain different shapes under controlled growth conditions [152,153,154], it was found that the uniform size of noble metal nanoparticles could be obtained in the microreactor systems. The microreactor allows control of the process of synthesis by separating each reaction step during nanoparticle formation, i.e., reduction, nucleation (1) and growth (2). In this way, substrates, intermediates and products can be separated simply (Figure 9).

Moreover, given in this section, the synthesis procedure shows the strong influence of the chosen flow rate on the size of obtained particles. Silver nanoparticles are a good example, characterized by high conductivity and biocompatibility. They have found applications in electrochemical sensors, structural materials and others. In addition, AgNPs have antimicrobial, anti-inflammatory and skin-tissue regenerating properties, enabling their wide application in medicine, pharmacy and cosmetology [155,156]. Silver nanoparticles are mainly obtained by chemical methods and also by less-common physical and biological methods [157]. However, a method currently gaining popularity for producing silver nanoparticles consists of the use of flow microreactor systems.

**Figure 10:**
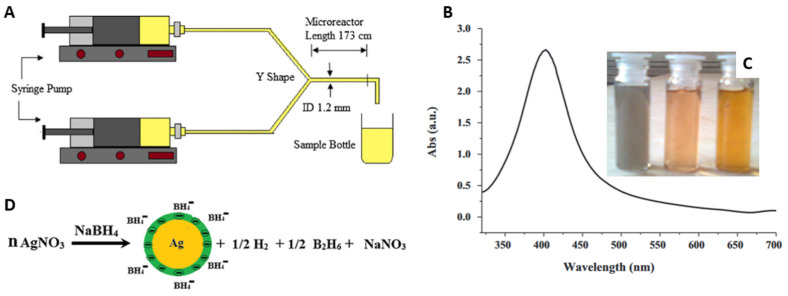
(**A**) Schematic diagram of the experimental setup for the synthesis of silver nanoparticles in microreactor. (**D**) Reduction reaction to generate silver colloidal particles synthesized in the presence of surfactant. (**B**) UV absorption spectrum of clear yellow colloidal Ag in a microreactor. (**C**) Colloidal silver in different stages of formation: grayish precipitate without surfactant, clear yellow sol at initial stage using surfactant and dark yellow sol at final stage using surfactant (AgNO_3_ flow rate = 1 mL/min, NaBH_4_ flow rate = 3 mL/min, space time = 0.48 min). (For interpretation of the references to color in this figure legend, the reader is referred to the web version of the paper). Reprinted from [158], Copyright (2024), with permission from Elsevier.

Patil et al. [158] examined the influence of surfactants on the synthesis of silver nanoparticles in a continuous flow microreactor. The synthesis was carried out in a spiral microreactor fabricated from low-density polyethylene (LLDPE). Two syringe pumps were inserted in the microreactor, making a Y-shaped geometry (Figure 10A). The precursor of the silver ions was silver nitrate (AgNO_3_). As a reductant, they used sodium borohydride. To control the size of the nanoparticles, two surfactants, sodium dodecyl sulfate (SDS) and CTAB, were used. The optimal synthesis conditions were determined at a flow rate of 1 mL/min AgNO_3_ (0.0001 M) and 3 mL/min NaBH_4_ (0.0003 M). The absorption spectrum of clear yellow colloidal Ag in the microreactor is shown in Figure 10B. The silver nanoparticles obtained in the microreactor had spherical shapes and smaller sizes (10–14 nm) than those obtained in the batch reactor (35–50 nm). The addition of the surfactant SDS reduced the size of the nanoparticles to about 3–7 nm. The size of the silver nanoparticles synthesized with CTAB in a microreactor showed a wide size distribution (from 4 nm to 18 nm). Figure 10C shows the colloidal silver in different stages of formation: a grayish precipitate without surfactant, a clear yellow sol at the initial stage using surfactant and a dark yellow sol at the final stage using surfactant. Figure 10D illustrates the reduction reaction that generates silver colloidal particles in the presence of surfactant.

The effect of segmented flow on the size of obtained silver nanoparticles was studied by Kumar et al. [159]. In the experiment, they used a spiral capillary microreactor. The synthesis was carried out in liquid–liquid and liquid–gas phases. In both cases, the aqueous phase was AgNO_3_–SASL, and an inert phase was kerosene (liquid–liquid phase) and air (liquid–gas phase). The reactions were carried out at a temperature of 90 °C, different residence times 10.5, 105 and 300 s and respective flow rates 1, 100 and 35 mL/min. The aqueous-to-inert phase ratios were 1:0.5, 1:1 and 1:2 in the liquid–liquid phase and 1:1, 1:2 and 2:1 in the liquid–gas phase. During the reaction of the two liquids, kerosene forms a continuous phase due to the smaller wetting angle with PMMA, and the aqueous phase is in the form of dispersed barrels. However, in the gas–liquid reaction, AgNO_3_-SASL is the continuous phase, while air is the dispersive phase. In the gas–liquid reaction, the obtained silver nanoparticles were smaller in size than in the case of liquid–liquid synthesis. The synthesis of silver nanoparticles using microdroplets as a reactor was carried out by Wojnicki et al. [160]. The metal precursor was silver nitrate and the reductant was 97% dimethylamine borate complex. The oil (heptane)-phase flow rate was 5 mL/h, and the aqueous-phase flow rate was 1 mL/h. The surfactants and stabilizers were PVA and Tween 80, respectively. To compare the results, the synthesis with the same conditions (temperature, concentration of reductant and precursor and addition of stabilizers) was carried out in a batch reactor. The use of microdroplets was shown to reduce the size from 4.8 ± 1.3 nm to 2.5 ± 0.5 nm, in comparison to the batch reactor.

Horikoshi et al. [161] used a hybrid flow microreactor to produce small silver nanoparticles. As a metal precursor, the [Ag(NH_3_)_2_]^+^ complex was used, whereas glucose and PVP were used as a reducing agent and particle stabilizer, respectively. The hybrid microreactor consisted of a chip-based microreactor, which provided good mixing of reagents. During the process, the reactants were introduced into the quartz spiral reactor combined with a microwave generator (the flow rate was 1 mL/min) as the source of the heat. Such a combination gave the possibility to produce small silver nanoparticles of about 4 nm in size. The study was also carried out with conventional stirring using microwaves, but nanoparticles were not formed.

**Figure 11:**
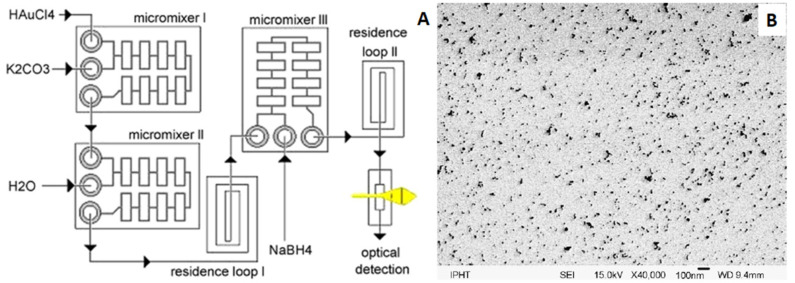
Modular microreactor arrangement for flow-through process: (**A**) Synthesis of gold nanoparticles. Examples of prepared gold nanoparticles (SEM analysis) (**B**). Reprinted from [165], Copyright (2024), with permission from Elsevier.

The next considered metal is gold. Gold nanoparticles have a wide range of applications, including medical (drug delivery, photothermal therapy, imaging and antimicrobial agents), water purification, food industry and biological applications [162]. Gold nanoparticles are also used in biosensors due to their physicochemical properties, ease of synthesis, and optical properties that vary with size and shape [163]. In recent years, microreactors have been successfully used for gold nanoparticle synthesis. Wagner et al. [164] used a continuous flow microreactor to produce gold nanoparticles. The microreactor was a three-layer chip-shaped arrangement of wet-etched Pyrex glass and silicon, and had eight splits and recombinant units to intensify mixing. In addition, hydrophilization of the microchannels was used to inhibit reactor channel clogging. The gold precursor was HAuCl_4_ (1 mM), whereas 20 mM of ascorbic acid was used as the reducing agent. Moreover, various amounts of PVP were added in order to stabilize particles. The solutions containing these reagents were pumped into a micromixer at flow rates between 500 and 8000 μL/min. Wagner et al. [165] also used a microfluidic system to produce small gold nanoparticles by reducing chloroauric acid with NaBH_4_. During this synthesis, microreactors containing eight splits and recombining units were used (Figure 11A). They show that synthesis in the microreactor yielded gold nanoparticles with reproducible sizes in the range of 4 to 7 nm (Figure 11B).

**Figure 12:**
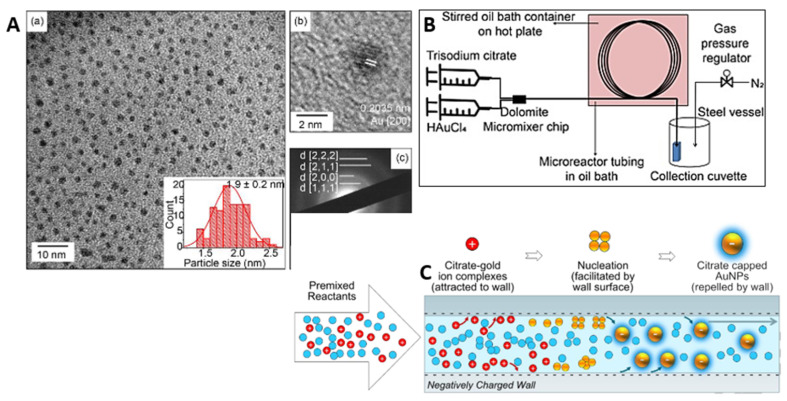
(**A**) (a) TEM images of gold nanoparticles obtained at flow rate of 0.006 mL/min and at 100 °C. (b) The single gold nanoparticle with the inter-planar spacing in the [2 0 0] direction. (c) SAED pattern of the gold nanoparticles synthesized. Solutions of 0.54 mM HAuCl4 and 1.7 mM citrate were mixed at 1/1 volumetric flow rate ratio. (**B**) Experimental setup; (**C**) interaction between the reactants and the microreactor wall. Reprinted licensed under Creative Commons CC BY from [167].

The synthesis of ultrafine colloidal gold nanoparticles of less than 5 nm in size was described by Sen et al. [166]. In the experiments, two variants of continuous and segmented gas–liquid flow were used. The microreactor was a PTFE capillary immersed in a hot oil bath. In the case of continuous flow, the gold precursor HAuCl_4_ was mixed with trisodium citrate and a stabilizing agent (PVP) was added using a syringe pump into the microcapillary. In turn, two syringe pumps were used in the segmented flow. One was used to add the mixed solution, while the other was used to infuse air. It was noted after the experiment that smaller and more monodisperse nanoparticles were produced in the presence of PVP. Moreover, lower flow velocities lead to the formation of smaller AuNPs. Smaller and more monodispersed particles were also formed at higher reduction temperatures. The use of segmented flow (gas) reduced the average particle size (below 5 nm) and polydispersity. Huang et al. [167] synthesized ultra-small gold nanoparticles using a continuous flow capillary microreactor system. The microreactor system, shown in Figure 12B, consisted of a PTFE capillary tube with an inner diameter of 0.3 mm, operating at a flow rate of 0.006 mL/min, an average residence time of 30 min and a temperature of 100 °C. Gold(III) chloride trihydrate and trisodium citrate were used as reactants. The negatively charged walls of the capillary facilitated enhanced nucleation by attracting positively charged gold citrate ion complexes (Figure 12C). This process led to the formation of gold nanoparticles with an average size of 1.9 ± 0.2 nm. Figure 12A contains a TEM image showing the dispersed nanoparticles (a) and a crystal lattice image showing the homogeneity and small size of the nanoparticles (b). The citrate-coated nanoparticles showed better SERS performance due to the weaker binding strength of the citrate ligand, which allowed for the easier adsorption of analyte molecules.

**Figure 13:**
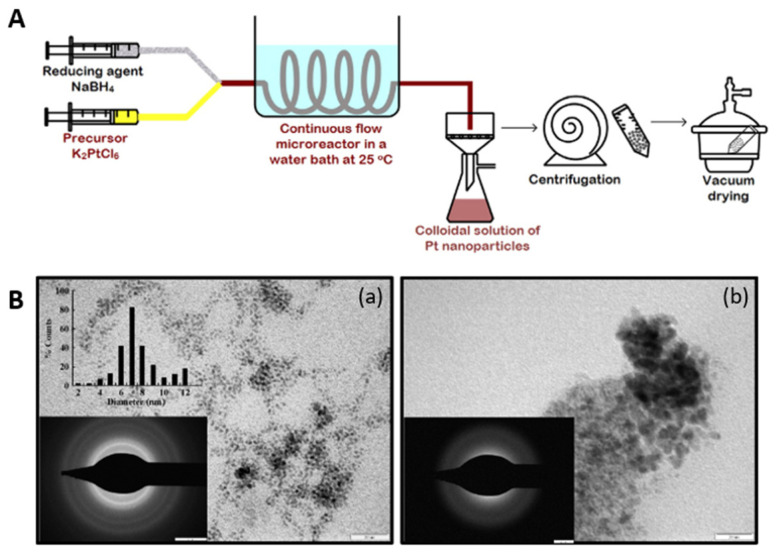
Experimental setup for synthesis of Pt nanoparticles in a continuous flow microreactor (**A**). Effect of flow rate in continuous flow microreactor on the nanoparticle size and crystallinity as observed by TEM and SAED. (**B**) (a) Flow rate = 14 μL/s and (b) flow rate = 28 μL/s (image scale: both TEM 20 nm, both SAED 5 nm). The inset histogram shows the size distribution of particles synthesized at flow rate 14 μL/s. Reprinted from [169], Copyright (2023), with permission from Elsevier.

Another example is platinum, whose nanoparticles are used in various fields, mainly in catalysis, pharmaceuticals, cosmetology and biomedical applications. The main catalytic properties of platinum nanoparticles depend on their morphology [168]. Flow microreactor technology makes it possible to synthesize platinum nanoparticles (PtNPs) of specific shapes and sizes. Suryawanshi et al. [169] used a continuous flow microreactor to synthesize ultra-small platinum nanoparticles. As the metal precursor, potassium hexachloroplatinate was used, while sodium borohydride and PVP were used as a reducing and stabilizing agent, respectively. The synthesis was carried out in a spiral microreactor at 20 °C (Figure 13A). The flow rate was maintained at either 14 or 28 μL/s. In the experiments, the flow rate had a significant influence on the size of the platinum nanoparticles. It was shown that at higher flow rate, nanoparticles were aggregated and reached a size of 15 nm (Figure 13B(a)). At a lower flow rate, 7 nm particles were obtained (Figure 13B(b)).

Platinum nanoparticles were synthesis in one microreactor cycle by Luty-Błocho et al. [170]. In this case, as a platinum precursor, H_2_PtCl_6_ was used. Sodium borohydride dissolved into sodium hydroxide was used as a reducing agent. The synthesis of the catalyst was carried out either with the addition of or without stabilizers (PVA and PVP) at a flow rate 2 mL/min and 4 mL/min, at room temperature 20 °C. By reducing Pt(IV) ions in the presence of sodium borohydrate (NaBH_4_) in a microreactor, PtNPs with sizes ranging from 3.5 nm to 5 nm were formed, depending on the flow rate of the reactants. At a flow rate of 2.0 mL/min, PtNPs with a size of 5.0 ± 1.2 nm were obtained, while a flow rate of 4.0 mL/min yielded smaller nanoparticles with an average size of 3.5 ± 1.5 nm. The process showed high deposition efficiency on ACF (Activated Carbon Fibers), from 81% to 95%.

Riche et al. [171] synthesized platinum nanoparticles in a droplet microreactor. PtNPs were synthesized using a combination of a H_2_PtCl_6_ stream and ionic liquid droplets as the dispersed phase and reaction medium. The ionic liquid droplets could be recycled and reused to produce PtNPs. The reaction was carried out at 80 °C and a flow rate of 1.5 mL/h, with a reaction time of 30 min. The formed PtNPs were 2–3 nm in size with the yield of the process around 60%. This efficiency was almost doubled compared to the synthesis carried out in the batch reaction.

Palladium nanoparticles are a valued material due to their chemical and thermal stability, as well as their electrical or optical properties [172]. Palladium is also the second element most frequently used in catalysis. The synthesis of PdNPs with controlled size and shape is important to obtain good catalytic properties [173]. The size of nanoparticles in a batch reactor can be controlled by using ligands or stabilizers that adsorb on metal surface [174,175]. Currently, the size of palladium nanoparticles can be controlled by varying process parameters in systems using a flow microreactor. The synthesis of palladium nanoparticles of different sizes was carried out by Sharada et al. [176] using a copper continuous flow microreactor. The precursor for the synthesis of PdNPs was palladium (II) chloride (PdCl_2_); as a reductant, they used sodium borohydride and CTAB was used as a surfactant. The microreactor was immersed in a bath to maintain a temperature of 25 °C. The process was carried out at different flow rates between 30 and 50 mL/h. The final solution was centrifuged for 20 min at 8000 rpm and washed using acetone and deionized water to obtain PdNPs. The result shows that the size of palladium nanoparticles decreases with decreasing metal precursor concentrations from 150 nm to 50 nm. The smallest size of PdNPs was obtained for the concentration of reactants of 0.00025 M PdCl_2_ and 0.001 M NaBH_4_ and a flow rate of 30 mL/h.

### 4.2. Control of Nanomaterial Composition

Next to the control of the morphology of synthesized noble nanomaterials, there is a vision for creating more complex structures. This is usually realized by the modification of the basic procedure by adding to the known protocol of the synthesized second element. This affects particles properties, which can be adjustable by the kind of added compound and its amount. This approach results in a new class of materials which are known as a core–shell structure or bimetallic nanoparticles.

#### 4.2.1. Nanoshell (Core–Shell) Particle Production

**Figure 14:**
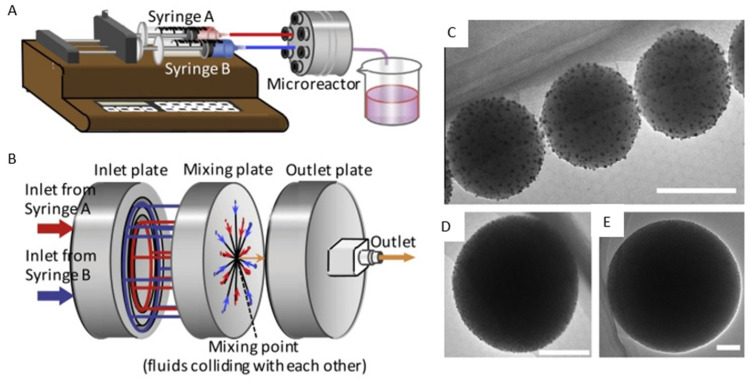
Scheme of the experimental setup. (**A**) Scheme of the central collision-type microreactor with three plates (**B**). The TEM images of particles after the AuNP: 120 nm core silica particles (**C**), 300 nm core silica particles (**D**) and 570 nm core silica particles (**E**). Reprinted from [180], Copyright (2024), with permission from Elsevier.

Nanoshells (core–shell nanomaterials) consist of a spherical core surrounded by a thin metallic coating (usually gold) about 1–20 nm thick [177]. The advantages of core–shell nanomaterials are that these materials can be synthesized with a desired chemical, electronic and photonic properties, as well as a specific structure and composition. The coating provides a protective, bio-compatible or inert surface [178]. Nanoshells are used in areas such as biosensors, drug delivery, gene screening, target therapy and cancer imaging [177,179]. Gold nanoshells are particularly attractive for biological applications since the metal is corrosion-resistant, has low toxicity and is a popular material in medicine. Watanabe et al. [180] produced gold nanoshells on modified silica particles in a central collision-type microreactor with three reaction plates (Figure 14B). The experimental setup is shown in Figure 14A. The reagents were an APTS-SiO_2_ suspension containing HAuCl_4_ (0.075 mmol/L) and NaBH_4_ (1.5 mmol/L), which were introduced to the system using syringe pumps. The total flow rate was 20 mL/min. The experiments were carried out at room temperature. The gold-seeded silica particle suspension containing gold ions in the shell were mixed with ascorbic acid (reducing agent) in the microreactor. The experiment was focused on the influence of core sizes (120 nm, 300 nm, 570 nm) and types of reductant on the homogeneity of the shell and their morphology. The results showed that the key issue in gold nanoparticle deposition on a silica core was the appropriate ratio of the surface area of the silica core to the quantity of the AuNPs in the solution. The appropriate concentration of Au ions and the high mixing efficiency of the microreactor allowed core–shell clusters to be obtained on silica with a diameter of 120 nm (Figure 14C). Core–shell clusters were also obtained using core silica particles of 300 nm (Figure 14D) and 570 nm (Figure 14E), respectively. A constant surface area of the core particles was used. For both core diameters, there are uniformly deposited AuNPs on the silica particles. The result showed that the application of ascorbic acid caused the formation of non-homogeneous gold stains on the core surface. Using sodium citrate, single gold nanoparticles deposited on the silicon core were obtained.

Gomez et al. [181] fabricated SiO_2_-Au nanoshells in a three-stage microfluidic system. The microreactor system consisted of a PTFE tube and a micromixer immersed in a thermostatic bath (temperature control). The synthesis of the SiO_2_-Au nanoshells was carried out in a few steps. In the first stage of the process, the cores of the silica nanoparticles were formed. Solutions of ammonium hydroxide tetra-ethyl orthosilicate (TEOS) with 3-aminopropyltriethoxy-silane (APTES) were added to the system using syringe pumps (flow rate 5 mL/min). The reaction temperature was 40 °C. In the next step, Au-seeded silica nanoparticles were obtained using an analogous microreactor system and reaction conditions. In this step, gold nanoparticle seeds (2–4 nm) attached to the amino-functionalized silica surface were obtained. The final step involved the growth of a coating of Au nanograins deposited on silica nanoparticles. Formaldehyde was used as the reduction agent. Au-seeded silica shells were obtained due to the careful control of the reaction conditions, and particularly the pH (2.5, 6.5). Nanoshells with suitable plasmonic properties were obtained. Moreover, high reproducibility of the product was achieved. The analogous synthesis process carried out in the batch reactor was much longer and consumed more reagents.

The core–shell nanoparticles can also be silicon-core silver coatings. The colloidal silver particles have a stronger plasmon resonance band than gold particles of the same size. Meincke et al. [182] prepared a core–shell coating of silver nanoparticles on colloidal silica particles using a continuous-flow microreactor as a static T-mixer. The mixer was made of stainless steel (system 1) and polyamide tubes (system 2). In the system, glass syringes were connected to coiled stainless steel tubes placed in a water bath on a heating plate. A dilute aqueous solution of ammonia (less than 60 mM), silver nitrate (100 µM to 550 µM) and formaldehyde (less than 0.5%) was used. Reagent flow rates ranging from 10 mL/min to 100 mL/min were used. At a flow rate of 16 mL/min, heterogeneous particles were obtained, with spots covering about half of the silica core. Increasing the flow rate to 100 mL/min (system 2) resulted in the formation of nearly complete silver coatings at higher concentrations of silver nitrate (450 µM). Maw et al. [183] presented a successful mechanism for the synthesis of homogeneous and smooth silver nanoshells on silica cores. They used a central collision-type microreactor due to its high efficiency in mixing reagents. In the experiment, the impact of several agents to create a stable shell was analyzed. The kind of reductant (ascorbic acid or formaldehyde), the impact of stabilization and the ratio of the amount of ammonia to silver ions were taken into account. The complete silver nanoshells on the SiO_2_ core were obtained using formaldehyde (weaker reducer) and at a ratio of the amount of ammonia to silver ions around 1000. The addition of PVA, besides stabilizing the suspension, also supported the growth of the silver shell. Kalele et al. [184] used a microreactor to synthesize silver nanocoatings for the rapid detection of Escherichia coli. The synthesis involved coating 200 nm silica particles with silver coatings approximately 20 nm thick. Synthesis parameters included the use of silver nitrate reduced with trisodium citrate and the controlled growth and incubation of E. coli in Luria–Bertini broth. The final products showed high specificity and selectivity in the presence of E. coli. The silver nanoparticles showed a shift in the surface plasmon resonance band from 443 nm to 458 nm. The particle morphology was spherical with an agglomeration of nanoparticles after antibody attachment. This configuration, using a controlled flow rate, facilitated the efficient and rapid optical detection of Escherichia coli.

**Figure 15:**
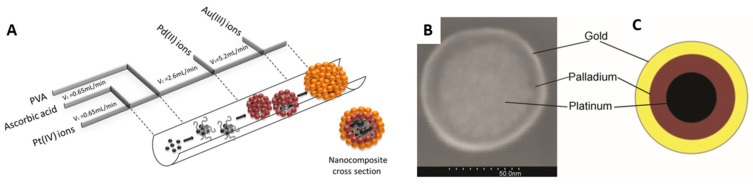
(**A**) Connection diagram of microreactors for nanocomposite synthesis, (**B**) TEM analysis of a single nanoparticle, (**C**) proposed structure of the nanoparticle. Reprinted from [185], Copyright (2024), with Springer Nature permission (license no. 5816621035376).

Luty-Błocho and Wojnicki [185] synthesized nanoparticles with onion-like structures containing three layers of different metals, i.e., platinum, palladium and gold (Figure 15). The process was carried out in a glass microreactor, which, schemed together with the location of inputs, is shown in Figure 15A.

The flow rates of reagents and temperature of the process were set based on kinetic data. It should be underlined that microreactor systems enable users to carry out the process at different temperatures. The application of a back pressure in the system allowed PtNPs to synthesize at 105 °C, and then the solution was cooled down to room temperature. After that, the streams containing Pd(II) and Au(III) ions were introduced to the microreactor channel (Figure 15A) and the efficient formation of metal layers occurred (Figure 15B,C).

Knauer et al. [186] used a microfluidic system to synthesize Au/Ag core/shell and Au/Ag/Au multishell nanoparticles using a segmented flow method. Their microreactor used three syringe pumps to introduce reactants into a PTFE reactor channel. The segmented flow was achieved by combining immiscible liquids, facilitating efficient mixing and a narrow residence-time distribution. Two immiscible phases were used to achieve segmented flow: an aqueous phase containing the reactants and a tetradecane carrier phase. The aqueous phase contained tetrachloroauric acid(III) (HAuCl_4_) and silver nitrate as metal precursors; furthermore, ascorbic acid as a reducing agent and CTAB as a stabilizer were used. In the carrier phase, tetradecane is a non-reactive organic liquid. The synthesis process of Au/Ag core/shell nanoparticles required heating the microreactor to 80 °C, while the synthesis of Au/Ag/Au double-shell nanoparticles was carried out at room temperature. A concentration of 0.5 mM HAuCl4 was used for gold grains; the synthesis of Au/Ag core/shell nanoparticles required 10 mM AgNO_3_ and the synthesis of Au/Ag/Au double shells used 1 mM HAuCl_4_. The Au/Ag core/shell nanoparticles with a diameter of about 20 nm at a half distribution width of 3.8 nm, and Au/Ag/Au multishell nanoparticles with a diameter of about 46 nm at a half distribution width of 7.4 nm were obtained. The size and optical properties of these nanoparticles were dependent on the composition and thickness of the coating. The obtained nanoparticles can be used in sensors, bioanalysis and nonlinear optical devices.

#### 4.2.2. Bimetallic Particle Production

Kluitmann et al. [187] used an automated microfluidic system to synthesize bimetallic gold–platinum nanowires. The study used a computer-controlled syringe pump system to control the flow rate of the reactants and create a segmented flow. The microreactor configuration included a six-port manifold and a T-unit to create aqueous reaction segments in a continuous stream of perfluorinated oil. The process parameters included a total flow rate of 400 µL/min (250 µL/min for the carrier oil and 150 µL/min for the aqueous phase containing the reactants). The synthesis was carried out at an elevated temperature of 95 °C to ensure complete platinum deposition. Reagents involved in the synthesis included gold nanorods in CTAB solution (1.5 mM Au in 50 mM CTAB), sodium hydroxide (10 mM), potassium tetrachloroplatinate(II) (2.5 mM) and ascorbic acid (3 mM). The products obtained depended on the concentrations of the platinum precursor and reducing agent. At low concentrations of potassium tetrachloroplatinate(II) (0.1 mM), sparse platinum nuclei formed on the gold nanorods. When the concentration increased to 0.25 mM, the density of platinum nuclei increased. At the highest concentration (0.5 mM), a dense platinum film of about 17 nm in length and 3 nm in diameter was formed. The aspect ratio of the nanowires decreased from 4.5 to about 2.4 as the platinum concentration increased.

### 4.3. Catalyst Production

In recent years, catalysts based on noble metals have gained popularity due to their electronic and chemical properties [188]. Commonly used materials for catalysts include platinum and palladium. Rarely used catalysts include gold- and silver-based materials. Among the known materials for catalysts, bimetallic nanoparticles are an interesting option due to the synergistic properties of the two elements that make up the material [189]. In the case of palladium and platinum, low lattice mismatches facilitate bonding into alloys and core–shell structures, and their coexistence can enhance the catalytic activity of the material [190]. Nanoparticle morphology has been shown to strongly influence the activity and selectivity of catalytic reactions [191,192]. The synthesis of materials in microreactors offers the possibility of precisely tailoring the shape and size of nanoparticles using controlled synthesis. Furthermore, it is possible to improve catalytic performance while reducing the cost of the material.

Besides nanoparticle morphology, the catalytic properties also depend on the catalyst support [193]. The support materials used are mesoporous and microporous, and in the case of Pt and Pd, they can be activated carbon [194], TiO_2_ [195] and silica [196,197].

**Figure 16:**
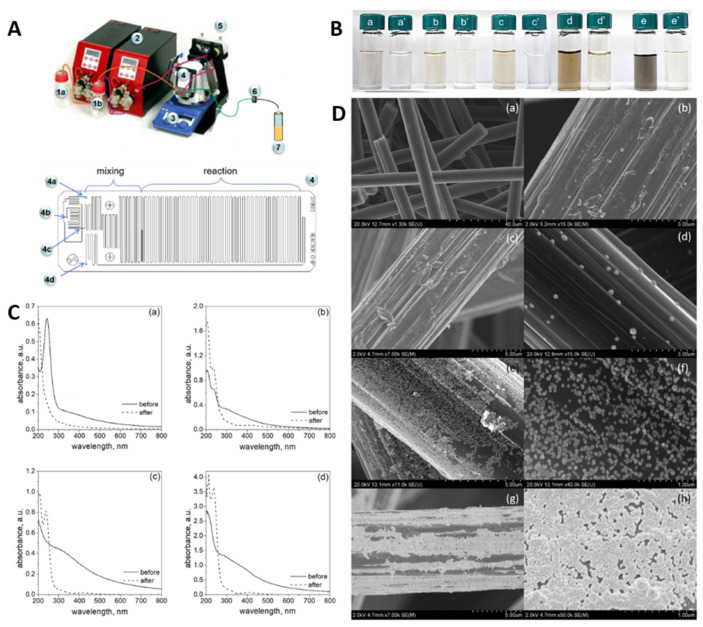
(**A**) The scheme of microreactor system used for continuous PdNP synthesis and their deposition on activated carbon fibers. 1a—A reservoir for the reducer, 1b—water, 2—a pump, 3—a thermostat, 4—a microchip, 4a—reductant input, 4b—metal precursor input, 4c—double T-mixing junction, 4d—fluid output, 5—a reservoir for Pd(II) ions, 6—filter with active carbon fibers, 7—sample. (**B**) Colors of obtained solutions containing palladium nanoparticles resulting from the reduction of the chloride complex of Pd (II) with L-ascorbic acid. Conditions: C_0,Pd(II)_ = 0.05–0.4 mM, C_0,AA_ = 0.1 (a)–0.8 mM (e′), *T* = 40 °C, *I* = 0.0 M, C_0,Cl¯_ = 0.0 M. (a–e) denote sample before solution passing through filter with ACF, and (a′–e′) after solution passing through filter; (**C**) UV–Vis spectrum of colloidal phase of palladium obtained as a result of reduction reaction of Pd(II) ions with L-ascorbic acid. Conditions: FR = 6.0 mL/min, *T* = 40 °C, *I* = 0.0 M, C_0,Cl¯_ = 0.0 M, C_0,Pd(II)_ = 0.05 mM, C_0,AA_ = 0.1 mM (a). C_0,Pd(II)_ = 0.1 mM, C_0,AA_ = 0.2 mM (b). C_0,Pd(II)_ = 0.2 mM, C_0,AA_ = 0.4 mM (c). C_0,Pd(II)_ = 0.3 mM, C_0,AA_ = 0.6 mM (d); (**D**) SEM analysis: (a) Activated carbon fibers, (b–h) Pd/ACF catalyst obtained as a result of nanoparticle synthesis in microreactor and their deposition on activated carbon fibers. Conditions: (a) FR = 6.0 mL/min, *T* = 40 °C, *I* = 0.0 M, C_0,Cl¯_ = 0.0 M and (b) C_0,Pd(II)_ = 0.05 mM, C_0,AA_ = 0.1 mM, (c) C_0,Pd(II)_ = 0.1 mM, C_0,AA_ = 0.2 mM, (d) C_0,Pd(II)_ = 0.2 mM, C_0,AA_ = 0.4 mM, (e,f) C_0,Pd(II)_ = 0.3 mM, C_0,AA_ = 0.6 mM, (g,h) C_0,Pd(II)_ = 0.4 mM, C_0,AA_ = 0.8 mM. Reprinted and licensed under Creative Commons CC BY from ref. [200].

Federici et al. [198] carried out the combustion of synthesis gas (a mixture of CO and H_2_) on a Pt/ Al_2_O_3_ catalyst. The supported catalyst was synthesized by wet impregnation. The process used a microreactor made of two thick stainless steel walls. Inside the microreactor, there was a thin stainless steel plate with a 450 μm groove for placing the catalyst. The Pt/Al_2_O_3_ catalyst was placed between a fibrous alumina mesh to hold the bed in place. The catalyst plates were placed adjacent to the inlet and outlet of the microreactor. The total flow rate was maintained at 200 Nm/min. The pressure in the system varied from 1.7 to 1.9 atm. Reactions were carried out at H_2_-to-CO ratios similar to coal gasification and methane reforming. It was found that CO significantly inhibits the oxidation of H_2_. In contrast, the addition of H_2_ promotes CO oxidation at low molar fractions. The carrier for the Pt and Pd catalysts can also consist of activated carbon fibers. Luty-Blocho et al. [170] developed a process for platinum deposition on activated carbon fibers involving the use of a microreactor system. The reaction system consisted of a mixture of an aqueous solution of Pt(IV) ions and NaBH_4_. After the reduction of the platinum ions, a polymer was added to the system to prevent the further growth of the platinum particles. The resulting solution stream consisted of stable particles that flowed through a PTFE capillary into an ACF-filled filter. The microreactor system allowed proper mixing of reagents and efficient deposition of platinum nanoparticles directly on the ACFs. Platinum nanoparticles synthesized in the microreactor were characterized by transmission electron microscopy (TEM) and X-ray diffraction (XRD). The TEM images showed that the platinum nanoparticles had a spherical shape and an average size of 3.5 nm. Luty-Blocho et al. also performed gold [199] and palladium [200] deposition on activated carbon fibers in a microreactor system (Figure 16A).

The metal precursor was mixed and reduced with L-ascorbic acid in microreactor (Figure 16A). Then synthesized particles were deposited on activated carbon fibers (ACF as the PdNP carrier) placed in a filter. Colloidal palladium with different colors (Figure 16B) were synthesized under various conditions, and the optimal resistance time. Total flow rate in the microreactor were determined from kinetic data obtained in the previous study. Tests conducted on the final material showed that the PdNPs were successfully deposited on the carbon substrate (Figure 16D). Process parameters, such as the initial concentration of metal ions and reductant, were found to affect the size and shape of the PdNPs produced. Overall, the method of depositing PdNPs on a carbon substrate in a microreactor system proved to be successful in producing nanoparticles of controlled sizes and shapes with high efficiency.

**Figure 17:**
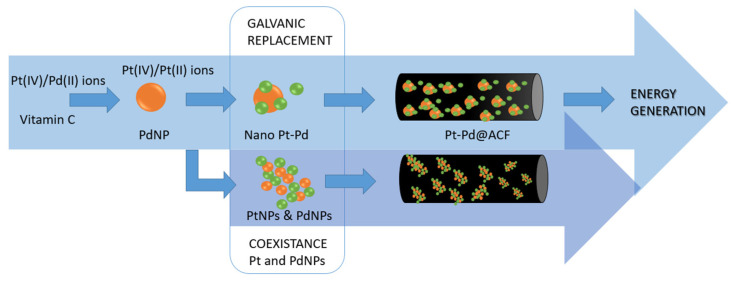
Schematic representation of the synthesis process for the Pt−Pd@ACF catalyst in a microreactor system. The process begins with the mixing of Pt(IV)/Pd(II) ions and ascorbic acid (Vitamin C), leading to the formation of Pd nanoparticles. These nanoparticles undergo galvanic replacement and coexistence to form nano-Pt-Pd and Pt-Pd nanoparticles. The nanoparticles are then deposited onto activated carbon fibers (ACFs), resulting in the Pt−Pd@ACF catalyst, which is utilized for energy generation. Reprinted and licensed under Creative Commons CC BY from [201].

Pach et al. [201] demonstrated a one-step synthesis of a bimetallic Pt−Pd@ACF (ACFs—activated carbon fibers as the catalyst carrier) catalyst using a microreactor system for the hydrogen evolution reaction (HER). The study employed a glass microreactor that facilitated the reduction of Pd(II) and Pt(IV) ions in the presence of ascorbic acid, resulting in the formation of Pt−Pd nanoparticles. Depending on the flow rate, the composition and size of the nanoparticles varied. At a flow rate of 5 mL/min, the catalyst comprised 32% Pt and 68% Pd. The microreactor system proved to be more efficient for catalyst synthesis compared to conventional batch reactors, producing catalysts with better dispersion and higher catalytic activity. This process, illustrated in the accompanying Figure 17, highlights the steps from the initial mixing of Pt(IV)/Pd(II) ions and ascorbic acid, through galvanic replacement and the coexistence of Pt and PdNPs, to the final deposition on the ACFs and the generation of energy.

Noble metal catalysts can exist in microreactors in the form of a packed bed. The packed-bed reactors are filled with solid catalyst particles and the chemical reaction proceeds on the surface of the catalyst. This design is most often used to catalyze gas reactions. The advantage of this averment is the higher conversion per weight of the catalyst than in other catalytic reactors [202]. Pekkari et al. [203] worked at a synthesis method using a continuous flow microreactor to synthesize Pd nanosheets and PdPt core–shell nanoparticles (Pd with dendritic Pt coating). Catalytic tests were carried out for the reduction of NO_2_ in the presence of H_2_ in a pocket reactor. A pocket microreactor is a quartz tube inside which there is a catalyst sample holder, consisting of a quartz pocket. Pd nanocages and PdPt core–shell nanoparticles exhibited high catalytic activity at low temperatures (<136 °C) and exhibited thermal stability. In addition, they showed high selectivity toward N_2_ and N_2_O formation compared to Pd nanosheets.

### 4.4. Synthesis under Special Conditions

In many applications, especially in medicine and catalysis, important aspects relate to particle compatibility. This can be reached by the use of an aqueous base solution containing nanoparticles. Sometimes, to enhance the process, the synthesis requires a high temperature, which is difficult to achieve at standard synthesis protocol.

***Silver***. The silver nanoparticles were synthesized by Yang et al. [204] in a continuous-flow tubular microreactor. Silver pentafluoroprionate (as a metal precursor) was thermally (at 100–140 °C) reduced in isoamyl ether to form silver nanoparticles in the presence of trioctylamine. In this synthesis, a flow rate was in the range from 0.08 mL/min to 0.7 mL/min. Ag nanoparticles precipitated from the solution using ethanol and after centrifuging for 5 min at 5000 rpm. The synthesis conducted at the flow rate of 0.008 mL/min produced silver nanoparticles with the size 8.7 ± 0.9 nm. Increasing the flow rate to 0.6 mL/min yields polydisperse nanoparticles, and their size was between 3 and 12 nm. Lin et al. [205] synthesized monodispersed silver nanoparticles in a continuous-flow stainless steel tubular microreactor. The reactants, silver pentafluoropropionate and trioctylamine (molar ratio 3:1), were dissolved in isoamyl ether and introduced into the system using a syringe pump. The flow rate was 0.08 to 0.7 mL/min. The capillary microreactor was immersed in an oil bath whose temperature was kept between 100 and 140 °C. It was shown that the flow rate and temperature of the reactor could significantly affect the size and size distribution of Ag nanoparticles. At 120 °C, nanoparticles of 8.3 ± 1.2 nm were obtained, while the process at 140 °C yielded nanoparticles of 7.4 ± 1.4 nm. Zhang et al. [206] synthesized silver nanocrystals in a droplet microreactor separated by air. The experiment gave silver nanocrystals of various shapes (nanocubes and octahedra) and sizes by controlling the flow rate, reaction time, concentration of reagents and temperature of the synthesis. The droplet microreactor was fabricated from PTFE tubes and two silica capillaries. Moreover, mixing zones 100 mm long were introduced. The optimal synthesis conditions were determined for a flow rate for reactants of 25 μL/min and for oxygen 30 μL/min, and a temperature of 140 °C. Silver nanocubes were obtained using single-crystal cubic silver. Various nanocube sizes were obtained for different reaction times, amounts of silver single-crystals and concentrations of AgNO_3_. Increasing the concentration of AgNO_3_ led to increased sizes of nanocubes (from 26 nm to 75 nm) obtained. Decreasing the amount of Ag seeds caused an increase in the edge length of nanocubes. Shu et al. [207] applied a droplet microreactor to synthesize water-soluble Ag_2_S quantum dots. The Ag_2_S nanocrystals are widely used as detectors, superionic conductors and photoconductive cells. Moreover, Ag_2_S can be used in fluorescent labeling for bioimaging in vivo. The synthesis was conducted in a droplet microreactor with PDMS fabricated using lithography. The liquid reactants containing an ethylene glycol disperse phase with MPA (115 mM) and AgNO_3_ (5 mM) and a soybean oil continuous phase were injected by syringe pumps in the microchannels. The flow rate was kept either at 20 μL/h or 10 μL/h. Synthesis was conducted at various temperatures from 81.8 to 117 °C. The reaction products were collected and centrifuged for 5 min at 12,000 rpm. During the synthesis of droplets, monodisperse nanoparticles of Ag_2_S were produced. The different sizes of Ag_2_S depended on temperature. At higher temperatures (110.7 °C), the size of the nanoparticles was smaller than at lower temperature (89.9 °C).

**Figure 18:**
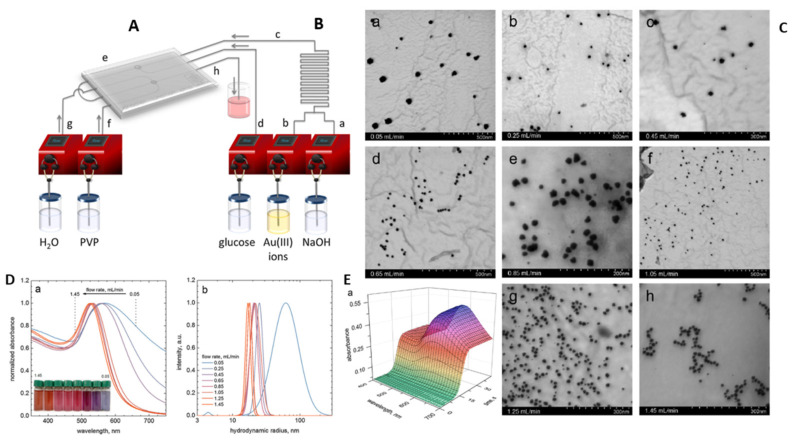
(**A**,**B**) The scheme of microreactor system used for AuNPs synthesis: a – channel with NaOH; b – channel with metal precursor; c – channel with the mixture of gold(III) and NaOH; d - channel with reductant (glucose); e – reactants after mixing, f – channel with stabilizer (PVP); g - channel with solvent (H_2_O); h – channel with gold nanoparticles solution (AuNPs). (**C**) TEM images of AuNPs obtained at different flow rates: (a) 0.05, (b) 0.25, (c) 0.45, (d) 0.65, (e) 0.85, (f) 1.05, (g) 1.25 and (h) 1.45 mL/min. (**D**) (a) Plasmon absorption bands and (b) hydrodynamic radius distribution of Au NPs obtained at different flow rates. (**E**) Time evolution of plasmon absorption band characteristic for AuNPs (λmax = 570 nm). Conditions: 0.15 mM Au(III), 1.5 mM glucose solution, T = 25 °C. Reprinted from [209], Copyright (2023), with permission from Elsevier.

***Gold***. Gold nanoparticles are very important materials due to their interesting physicochemical properties which depend on their size and shape. Obtaining appropriate gold nanoparticles requires a well-defined operating parameter such as the efficiency of mixing and sequence of addition of reagents. These parameters are difficult to obtain in conventional methods. The alternative method for obtaining gold nanoparticles is their synthesis in microreactor systems. The described technology allows conducting reactions using accurate process parameters [151]. Weng et al. [208] used a microfluidic system with microelectromechanical technology, integrating a micromixer, micropumps, microheaters, a microsensor and a micro-temperature valve on a single chip. The reactants were a 1 mM HAuCl_4_ solution and 38.8 mM of trisodium citrate. The reaction temperature was 115 °C. The successful synthesis of hexagonal gold nanoparticles was demonstrated. Usually, a spherical shape is observed in a conventional flask process. The formation of hexagonal gold nanoparticles in a shorter time was demonstrated. The process of noble metal nanoparticle synthesis in specific environment conditions undergoes hydrolysis. This, in turn, influences the form of the metal precursor and its effects on the final particles’ morphology. Pacławski et al. [209] carried out the synthesis of gold nanoparticles in a microreactor system, keeping under control the time of the hydrolysis of gold ions and particle morphology. To keep constant the time of the hydrolysis process in all experiments, the form of the metal precursor was the same. Next, [AuCl_3_(OH)]^−^ ions were reduced using glucose (Figure 18A,B). Such an approach provides an opportunity to control this step and finally enables obtaining particles with fine morphology.

Pacławski et al. [209] showed also that particle morphology can be adjusted by changing the amount of added PVP at constant flow rate. Alternatively, keeping a constant amount of polymer and changing only the flow rate of the stream containing the stabilizer, the size of the particles was adjusted. The diameter of the synthesized particles changed from 10 to 50 nm (Figure 18C(a–h)).

***Platinum***. Platinum nanoparticles, due to unique physicochemical properties [210], find applications mostly in catalysis, and recently in biomedical applications, including biosensors [211], cancer detection and treatment [212]. The process of their synthesis and choice of method (physical or chemical) depend on the subsequent application of particles. Thus, the choice of whether the synthesis will be carried out using a batch reactor or microreactor is forced by the conditions of the process in which they are to be used.

Pohar et al. [213] studied ethanol decomposition on a Pt/CeO_2_/ZrO_2_ catalyst in a microreactor made by low-temperature co-firing of LTCC ceramics. The catalytic beads were poured into the microreactor, then filled with fiberglass and sealed with graphite sealing tape. The packed-bed microreactor contained 2% Pt-CeO_2_ catalyst, deposited on spherical ZrO_2_ particles. Ethanol was passed through the system using glass syringes. The flow rate of liquid methanol was 2–70 µL/min. The temperature of the microreactor was maintained in the range of temperature 300–380 °C. Pt/Pd catalysts doped with cerium showed high catalytic activity. In addition, the presence of cerium lowered the temperature required for the complete conversion of methanol. Catalyst deactivation did not occur during the three months of experiments. A continuous flow microreactor system under high-temperature and high-pressure conditions was used by Luty-Blocho et al. [214]. The process of synthesizing platinum nanoparticles in the microreactor relied on the reduction of the chloride complex of platinum (IV) ions with ascorbic acid. The microreactor used in this study consisted of a chip and pumps, in addition to a thermostat, a back-pressure regulator and a sampler. The chip had a double T-mixing junction with a fluid input for platinum(IV) ions and polymer, and an input for ascorbic acid. The reagent’s container was connected to the chip by a PTFE microcapillary. A back pressure of 2 bar was used in the experiment to eliminate the boiling of the reagents in the system. The flow rate of the reagents was 2.4 mL/min, with the flow rates of individual reagents adjusted to achieve the desired reaction conditions. The synthesis of PtNPs in the microreactor took 6 s at 105 °C, compared to the batch reactor, where the process took 40 min at 40 °C. The resulting solution containing Pt nanoparticles was almost colorless and stable for at least 9 months. A synthesis of platinum nanoparticles characterized by various sizes and shapes was conducted by Sebastian et al. [215]. The synthesis was conducted in a segmented flow-based microreactor made of silicon. Nanostructures of different shapes (nanorods and nanosheets) were obtained using two gases, namely oxygen and carbon monoxide. The non-aqueous synthesis of Pt nanoparticles was conducted using platinum (II) acetylacetonate in mixtures of oleylamine and oleic acid. The platinum nanocrystals with shape dispersion (nanocubes, truncated nanocubes, nanorods, triangle nanoplates) were obtained at higher temperatures (>200 °C). The nanocubes were 5.6 nm in length. Moreover, the synthesis of platinum nanoparticles in an aqueous stream of K_2_PtCl_4_ segmented with CO required a residence time of about 150 s, whereas the hexagonal aggregates were obtained at 125 °C.

***Palladium***. A continuous flow reactor was used by Torigoe et al. [174]. It was a simple microreactor system consisting of a glass capillary tube and a syringe pump. The synthesis of palladium nanoparticles in the system occurred by thermal decomposition of palladium acetate in diphenyl ether. It takes place during the high-temperature decomposition of Pd(OAc)_2_ in the presence of poly(benzyl ether) dendrons as a stabilizer. By using a high temperature (>140 °C), the reaction does not require a reducing agent. The precursor solution was introduced to the system at a volumetric flow rate of 2.5–500 µL/ min. The capillary tubes were immersed in a silicone oil bath. The temperature of the bath was changed from 140 to 200 °C. The effect of reaction temperature and flow rate and the effect of the concentration ratio of the metal precursor and ligand were studied. It was found that the average particle size increased from 2.7 nm at 140 °C to 4.8 nm at 200 °C with increasing reaction temperature. The volumetric flow rate from 2.5 to 250 µL/min, at a constant capillary diameter of 320 µm and temperature of 180 °C, resulted in the particle size decreasing from 5.2 to 3.4 nm. A smaller particle size was obtained when the molar ratio of the ligand to the metal precursor was in the range of 1 to 5. The system yielded smaller particles at low ligand concentrations, which was not achieved in batch reactors. Sebastian et al. [216] used laminar and segmented flow to synthesize Pd nanowires. The synthesis was carried out in silicon/Pyrex chip microreactors designed to maintain a high temperature of 350 °C and pressure of 60 MPa. The reactor consisted of mixing, and reaction zones separated thermally. The temperature in the mixing zone was maintained at room temperature to avoid inhomogeneous nucleation, and the reaction zone was heated to 180 °C. The reactants were a palladium precursor (Na_2_PdCl_4_ + KBr + H_2_O) and polyol (EG and PVP). In continuous flow, the pressure was maintained at a constant of 0.8 MPa. In segmented flow, a gas stream of air and nitrogen was introduced into the microfluidic reactor. The gas/liquid volume flow ratios were modified from 0.8 to 3.5. Nanowires were obtained during synthesis in laminar flow at temperatures of 160 °C and 190 °C and a residence time of 120 s, while nanoparticles were formed at lower temperatures. The introduction of air into the system (segmented flow) improved the dispersion and allowed them to obtain Pd nanowires with a high aspect ratio. Increasing the temperature to 160 °C and 190 °C and pressure to 0.8 MPa allowed them to reduce the synthesis time to 2 min, while the batch process was much longer (1 h at 100 °C).

### 4.5. Challenges of Noble Metal Nanoparticle Synthesis in Microreactor Systems

**Figure 19:**
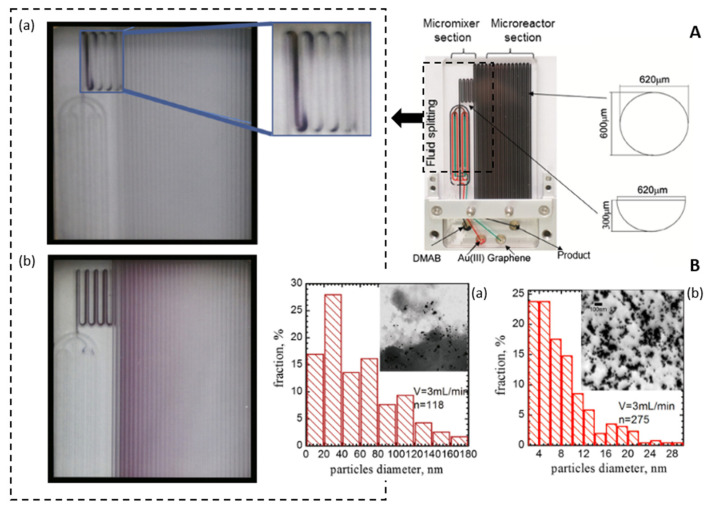
(**A**) Schematic arrangement of the channels (total volume 1 mL). The precipitation and adsorption of the metallic gold on the surface of microreactor channels at pH 13 (a) and at neutral environment (pH = 7) (b); (**B**) influence of the flow rate (V) on particle size and size distribution at pH = 13 (a) and 7 (b). Reprinted from [219], Copyright (2023) with permission from Elsevier.

One of the most common problems encountered during noble metal nanoparticle formation in the microreactor system is the uncontrollable process of particle deposition inside microreactor’s channel. This causes the formation of a metal layer on the wall and often its further autocatalytic growth. Ultimately, this leads to blocking of the hole in the microchannel, making synthesis impossible. The blocking of the channel basically appears during long-term synthesis and it results from consistent high roughness in the channel’s wall. The present microreactor technology allows for obtaining materials with lower porosity [217], but still the size of the pore (less than 100 nm) [218] is huge compared to the size of molecules or nanoparticles. The process of producing microreactors with walls of low porosity is much more expensive and does not fully work. The process of channel blocking was shown by Wojnicki et al. [219]. It was observed during gold nanoparticle synthesis and their deposition on graphene oxide sheets at pH = 7 and pH = 13 (Figure 19). To prevent channel blocking, a two-phase system was applied. In this procedure, an aqueous solution containing reagents was surrounded by oil to enhance synthesis efficiency and control nanoparticle size and distribution. The microreactor used in the study, as depicted in Figure 19A, consists of a micromixer and a microreactor section. The micromixer facilitates the initial mixing of reactants, while the microreactor provides the environment for the nanoparticle formation and deposition. Depending on the flow rate, the size and size distribution of the synthesized gold nanoparticles varied significantly. At a flow rate of 1 mL/min, smaller and more uniformly distributed nanoparticles were produced, as shown in Figure 19B(a). When flow rate increased to 3 mL/min, this resulted in larger nanoparticles with a broader size distribution (Figure 19B(b)).

A suitable material for microreactor constructions [220] can be high-quality glass with low porosity. Generally, the procedure of synthesis is fitted to existing systems. At present, the microreactors’ offer is within reach, but it still does not cover all the issues of nanoparticle synthesis, which require a specific system arrangement with few inlets in order to carry out more complicated synthesis. In order to reduce costs related to microreactor fabrication, mostly PTFE capillaries are introduced into the microreactor system. This solution is well known and is often applied. Depending on the role of the capillary, it can be connected to the microreactor channel in its various parts. For example, it may provide additionally substrates (stabilizer) to the reacting mixture at different times during the reaction progress. Sometime, a reaction is so long that more pronounced is using a long microcapillary instead of a microreactor. However, long-term synthesis might generate conditions for channel blocking.

**Figure 20:**
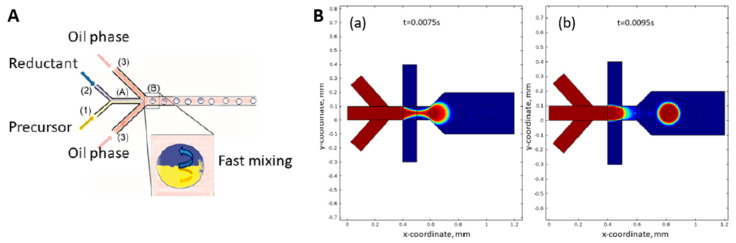
(**A**) Droplet microfluidic system including convective mixing inside droplet of a formerly bilayered reaction system. (1) The stream with metal precursor; (2) the stream with reductant; (3) oil phase stream; (**B**) simulation of droplet formation in the hydrophobic microreactor. (a) After 0.0075 s and (b) after 0.0095 s from the beginning of the experiment. Experimental conditions: V_H2O_,T = 1 mL/h, Voil,T = 2 mL/h. Reprinted from [221] under the terms and conditions of the Creative Commons Attribution (CC BY 4.0) license.

Another way to avoid clogging is to mix reagents in droplets. It is a promising way for efficient particle synthesis without the risk of microchannel blocking. Wojnicki et al. [221] used a microfluidic device to generate droplets containing mixed solutions of precursors and reductants. This method ensured efficient and uniform reactions in each droplet, resulting in nanoparticles with a narrow size distribution. In the study, the effects of different flow velocities and viscosities of the oil phase on droplet size and nanoparticle characteristics were verified. The microfluidic system presented separate inlets introducing streams of the precursor, reductant and oil phase. The precursor and reductant mixed before meeting the oil phase, forming droplets. This enabled the efficient synthesis of nanoparticles (Figure 20A). The CFD simulations of droplet formation at different time points illustrate the hydrodynamic focusing and initial pinching of the droplet in Figure 20B(a), while Figure 20B(b) shows the complete formation of the droplet. The microreactor system proved effective in producing consistent nanoparticles with minimal reagent consumption and waste generation. This method ensured a continuous synthesis process without clogging issues.

Choi et al. [222] demonstrated a microfluidic droplet-based system that was used to synthesize AuNPs. The main steps of the process included droplet generation, in which an aqueous solution of HAuCl_4_ and a mixture of NaBH_4_ with CTAB are introduced along with mineral oil to produce the droplets (i), synthesize AuNPs in the droplets (ii), and detect HRPs using the synthesized AuNPs (iii). The fluorescence image of the cross-channel microfluidic system showed the droplet formation process in detail, with a clear separation of the oil and aqueous phases of the solution, which is crucial for generation of uniform droplets and then nanoparticles. The AuNPs synthesized in the droplets had an average diameter of 16.69 ± 0.94 nm, while the particles synthesized in batches were larger and had an average diameter of 21.08 ± 0.80 nm.

**Figure 21:**
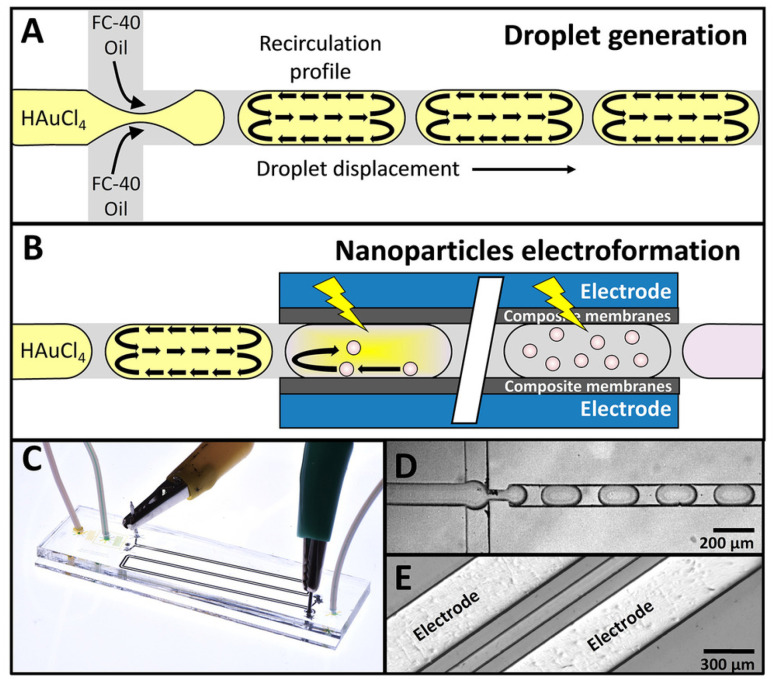
(**A**) Schematic illustration of the flow-focused approach for droplet generation. The recirculation flows inside the droplets are indicated with bold arrows. (**B**) After generation, the particles are sandwiched by two carbon-based membranes, which separate the droplets with gallium electrodes. (**C**) Photo of the microfluidic device. The power-supply electrodes are connected to the patterned gallium electrodes via two platinum wires. Micrographs of the microchannels for (**D**) droplet generation and (**E**) for the flow channel in between the pair of membranes and parallel electrodes. Reprinted from [224] under the terms and conditions of the Creative Commons CC-BY-NC license.

**Figure 22:**
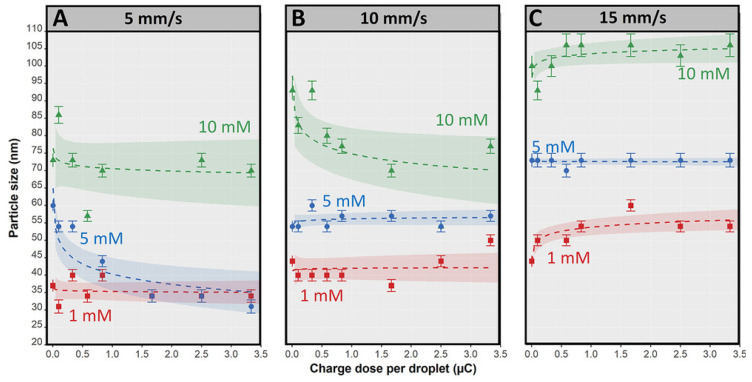
Particle size as a function of the charge dose per droplet and initial concentration of HAuCl_4_, for a droplet velocity of (**A**) 5 mm s^−1^, (**B**) 10 mm s^−1^ and (**C**) 15 mm s^−1^. Each point indicates an average particle size of >10,000 droplets. The shaded regions represent the 95% confidence intervals for logarithmic models fitted to the individual points. Reprinted from [224] under the terms and conditions of the Creative Commons CC-BY-NC license.

Abalde-Cela et al. [223] used a droplet microfluidic platform for the highly controlled and reproducible synthesis of branched AuNPs. Saucedo-Espinosa et al. [224] presented the electroformation of gold nanoparticles in droplets (Figure 21) The final size of the obtained nanoparticles depends on the initial concentration of the metal precursor and droplet velocity (Figure 22). The morphology can be modified by the change in pH. A microfluidic platform prevents particles from further aggregation through internal recirculation flows and the application of electrostatic stability.

Benyahia et al. [225] used the special construction of a microreactor system for introducing metal precursors in the stream of a reductant. Such a mixture was connected with the inverse stream of Miglyol 840 (Sasol GmbH, Hamburg, Germany), which is a propylene glycol diester of saturated fatty acids, primarily caprylic and capric acids. This approach enables the enclosure of reacting species by the surrounding oil phase. The microreactor setup facilitated the synthesis of gold nanoparticles (AuNPs) by mixing tetrachloroauric acid and ascorbic acid streams within monodispersed droplets, allowing precise control over the mixing efficiency and the size of the nanoparticles. The resulting AuNPs were tunable between 26 and 56 nm, depending on the premixing degree and internal mixing conditions of the reactants within the droplets. The computational fluid dynamics (CFD) simulations indicated a higher mixing efficiency at smaller droplet sizes, leading to reduced particle sizes.

Nie et al. [226] developed a spiral-shaped microchannel to facilitate the in situ patterning of silver nanoparticles, enhancing surface-enhanced Raman spectroscopy (SERS) for biomolecule detection. The microreactor design allowed for efficient mixing and uniform nanoparticle patterning, which are critical for achieving high-resolution SERS sensing. The examinations revealed that microchannel geometry significantly impacted nanoparticle size and uniformity. The optimal synthesis conditions included a silver nitrate concentration of 50 mM, an APTES-treated silicon substrate and a flow rate of 10 μL/min. These parameters produced silver nanoparticles with a hierarchical structure, providing substantial enhancement in Raman scattering. The microfluidic system effectively detected rhodamine B at concentrations as low as 1 pM and a 41-base single-stranded DNA sequence at 1 nM.

Another approach is the synthesis of metal nanoparticles carried out in a microreactor containing a hydrophobic surface. A hydrophobic surface can be obtained by a silanization process [227]. This process is carried out on a previously cleaned glass surface using plasma or wet chemistry treatment [228]. On the other hand, the silanization process can be used for further glass-surface modification. Rao et al. [229] synthesized a catalytic active gold-immobilized microchannel for ozonation. In this process, about 50% of the added pyruvic acid was decomposed within 0.75 s, confirming the performance of the device. In comparison, with the batch reactor, no decomposition of pyruvic acid was observed.

We hope that we managed to cover the majority of the papers concerned with nanoparticle formation in microreactors. Following our review of the existing literature, we have summarized in Table 4 the information about the synthesis of nanoparticles in microreactor systems.

## 5. Future Research Perspectives

Examples of microreactor applications in the process of nanoparticle synthesis collected in this review show that the subject of microreactor applications in nanoscience is not completely exploited. Some points should be raised which may be taken into account during investigations in the future. They are as follows:

1. The present state of the art indicates that the synthesis of nanoparticles is completed using a limited number of reducing agents, namely sodium borohydride, ascorbic acid, sodium citrate and glucose. Thus, the application of other reductants including bio-agents can be considered. Since the application of organic compounds may lead to the appearance of toxic substances, the use of microreactors (which are closed systems) may give better control over produced intermediates.
2. Mostly, water-based solutions have been used so far in the experiments. The organic phase appears only as a surrounding medium for water-based droplet formation. Consequently, one may use the agents (metal precursor and a reductant) in the organic solvent. Possible limitations consist of channel blocking, but gains resulting from better mixing properties of molecules dissolved in such a solvent should prevail.
3. The design of microreactors may progress by using new materials and more complicated architecture. Since the most common obstacles in their application are wall reaction and clogging, the creation of channel composite surfaces may be one possibility to avoid them. Another one can be inspired by nature, which may tell us how to transport the fluids upwards without obstacles. Moreover, the introduction of a gas (as is conducted in the case of bubble synthesis or segmented flow) may be a good solution for the application in multiphase systems.
  Also, attention should be turned to the analysis of the following:
  - The influence of back pressure, which allows high temperatures to be reached, preventing the boiling of water-based solutions. Temperature control over a wide range of results for better control of product morphology.
  - The introduction of multiphase systems (avoiding clogging) by introducing a gas. It gives an opportunity for nanoparticle synthesis in different environments. Solubility of different gases in the liquid medium may influence the mechanism and the rate of the reaction.
  - The functionalization of the nanoparticles can be assured by the modularity of a microreactor. The sequence of Y-type junctions will facilitate the control over subsequent stages of the reaction leading to the final product.
4. Investigations carried out with microreactor systems should be accompanied by kinetic studies. The rate of reactions (rate constants, activation energy) must be determined as an indispensable component of the reactor’s design.
5. A description of the process of nanoparticle formation requires a reliable model. However, such a model is still based more on authors’ imagination than on experimental information. New experimental tools are needed to provide better insights into the stages of the reaction proceeding from the appearance of the metal atom, through cluster growth, and finally to the formed particle.

All these problems mentioned above suggest that there is still a lot of work to do. Thus, the future for microreactors research seems to be bright.

## Figures and Tables

**Table 1:** The selected properties of metals (Ag, Au, Pd and Pt) at the bulk as well as at nanoscale. LSPR—localized surface plasmon resonance. Of note, colors given for the nanoscale relate to colors of colloidal particles mostly obtained using chemical methods.

Metal	Melting Temperature (K)	Optical Properties	Reference
Ag	1234	Metallic shine, gray tint (silver ^4^)	[2]
Nano Ag	898 ^1^	Yellow, orange, red, green, blue (LSPR)	[3,4,5]
Au	1336	Metallic shine, yellow (gold ^4^)	[6]
Nano Au	685–1115 ^2^	Orange, red, violet, blue (LSPR)	[7,8,9]
Pd	1828	Gray–white tint (palladium ^4^)	[2]
Nano Pd	above 500 ^3^	Dark orange, brown (LSPR in UV region)	[10]
Pt	2042	Pale grayish-white tint (platinum ^4^)	[11]
Nano Pt	above 800 ^3^	Gray, black (LSPR in UV region)	[12,13]

^1–4^ Of note, temperature values given in Table 1 relate to metals at nanoscale, and are example data coming from simulation study. ^1^ The value of temperature for 20 nm silver [14]; ^2^ 2.5–5.3 nm gold [15]; ^3^ 5 nm palladium and platinum nanoparticles [16]. ^4^ Silver, gold, palladium and platinum colors are defined in RGB (red, green, blue) model scale.

**Table 3:** Advantages and disadvantages of typical materials for manufacturing microreactors.

Material	Advantages	Disadvantages
Silicon	Widely available Low costs Good laminar flow even in multiphase reactions	Opaque Expensive manufacturing techniques
Glass	Transparent Structural stability Chemically resistant	Production constraints for complex projects Cracks under tension
Quartz	Excellent optical properties Resistant to high temperatures and pressure	High material costs
Metal and metal alloy	High heat and mass transfer properties Excellent mechanical strength	The high reactivity of certain metals
Polymers	Achieving complex structures with high precision on a small scale Mouldability (thermoplastics) Biocompatibility, flexibility, transparency (PDMS)	Low-temperature reactions required Incompatibility with certain solvents (PDMS) Low resistance to high pressure or temperature (PDMS)

**Table 4:** Synthesis of noble metal nanoparticles in the microreactor system using chemical approach.

Type of Metal	Chemicals *	Reacting Conditions **	Type of Microreactor	Flow Conditions	Particle Morphology	Findings	Ref.
Ag	0.1 mM AgNO_3_; 0.6 mM NaBH_4_ and 0.35 mM sodium citrate		3D curved microreactors			Quantitative analysis of the effect of mixing of precursors on the size and distribution of metal nanoparticles synthesized in flow microreactors.	[230]
	0.001 M AgNO_3_ and 0.002 M NaBH_4_		Polydimethylsiloxane (PDMS) microreactor				[231]
	50 mL of 1.0 mM aqueous AgNO_3_, Camphora lixivium	60 or 90 °C	Continuous flow tubular microreactors with glass and stainless steel	flow rate: 0.5 or 1.0 mL/min	Reactor A: quasi-spherical, size 11–40 nm; reactor B: quasi-spherical, size 12–38 nm; reactor C: quasi-spherical, size 9 to 33 nm	It has been shown that the polyols in lixivium are responsible for the reduction of silver ions. The process used three tubular microreactors with different internal diameters made of glass or stainless steel and different temperatures. At a temperature of 90 °C, silver nuclei exploded as a result of uniform nucleation, while at a temperature of 60 °C, polydisperse particles were formed.	[232]
	0.01–0.1 M AgNO_3_, PVP and AgNO_3_	170 °C, molar ratio of NaBH_4_ to AgNO_3_ was 0.7, molar ratio of PVP to AgNO_3_ was 0.05 to 1.5	Microchannel reactor made of acrylic resin	flow rate: 0.83 to 3.57 mL/s	130–13 nm	The influence of several variables on the obtained properties of AgNPs colloids in a continuous process was examined; the particle size was controlled by changing the weight ratio of PVP to AgNO_3_ from 0.05 to 1.5. The obtained silver nanoparticles were characterized by high purity and good crystalline structure.	[233]
	10 mM AgNO_3_, 1 or 25 mM CTAB, 10 or 1000 mM ascorbic acid		Straight and spiral millichannels	flow rate: 50 μL/min, reactors operated: 30 min	Spherical nanoparticles: 30–60 nm; triangular plates: 80–200 nm; nanorods: 130–150 nm	The influence of the ratio of reactants (silver nitrate and ascorbic acid) in batch, straight and spiral millifluidic reactors on the morphology of the synthesized silver nanoparticles was investigated. At higher reactant ratios, rod- and wire-shaped particles were obtained in straight and curved millifluidic reactors. At low reactant ratios, the particle shape is either triangular or rectangular in the three reactor configurations.	[234]
	0.06–0.45 mM AgNO_3_, 0.2–1.57 mM Na_3_CA×2H _2_O, 0.3–0.9 mM NaBH_4_	20 °C	Advanced-Flow^TM^ Reactor (AFR)	flow rate: 1–9 mL/min	7.2 ± 3.9 and 4.6 ± 1.8 nm (FR 1 and 9 mL/min); 7.5 ± 3.9 and 4.3 ± 1.8 nm (FR 0.25 and 8 mL/min).	As the total flow rate increased, the particle size distribution of Ag nanoparticles became narrower due to better micromixing efficiency at higher flow rates. When the flow rate ratio changed, the particle size distribution of Ag nanoparticles depended on the width of the Ag precursor solution.	[235]
	AgNO_3_, tannic acid, pH 10	20 to 45 °C, molar ratios of tannic acid/AgNO_3_: 0.5	Glass tubular reactor		14 to 60 nm	The effect of tannic acid concentration and temperature on the conversion of silver ions and the size of silver nanoparticles. The increase in temperature had a positive effect on the conversion rate of the resulting silver nanoparticles; it also affected the obtaining of nanoparticles with larger diameters.	[236]
Au	1.08 mM HAuCl_4_ and 5.4 mM trisodium citrate; 0.54 mM HAuCl_4_ and 1.7 mM trisodium citrate solutions	average residence time (1.5–30 min) and temperature (70–100 °C) 275 kPa back pressure,	Continuous flow capillary reactor		1.9–3.0 nm	Negatively charged capillary–solution interface offered enhanced nucleation rate; the size varied depending used flow rate and temperature.	[167]
	Seed gold nanoparticles: 10 mL of 0.5 mM HAuCl_4_ ×3H_2_O, 0.6 mL of 0.01 M NaBH_4_, 10 mL of 200 mM CTAB Microreactor: 4 mL of 1.25 mM gold salt, 4 mL of 250 mM CTAB, 0.05 mL to 0.25 mL of 4 mM AgNO_3_, 5.16 mM ascorbic acid	35 °C	Microfluidic T-junction.		Spherical–spheroidal, rod-shaped particles (about 2.3 ± 0.5, 3.2 ± 0.5, 4.0 ± 0.5, 2.7 ± 0.3) and sharp-edged AuNPs	The droplet-based microfluidic system (aqueous suspension of reactants and oil) for the production of anisotropic dispersions of Au nanocrystals. Au nanocrystals with different morphologies were synthesized by manipulating reagent concentrations and feed rates of individual water streams entering the microreactor.	[237]
	H_2_O + 1 mM HAuCl_4_ H_2_O_2_		Droplet–plasma microreactor	synthesis time: 120 μs	~4 nm	The method based on irradiating liquid droplets with ultra-low energy electrons (<0.1 eV) resulted in the rate of formation of nanoparticles in the plasma-droplet system exceeding other synthesis methods. Plasma droplet synthesis of small spherical Au nanoparticles has been demonstrated.	[238]
	1 mL 10 mM HAuCl_4_, 8.8 mL 0.05 M CTAB, 50 μL of a 40 mM AgNO_3_, 2.1 mM ascorbic acid, 60 µL NaBH_4_	30 °C	Continuous flow microfluidic chip	flow rates: 5 μL min^−1^, 3.6 μL min^−1^, 1 μL min^−1^, and 6 μL min^−1^, residence time-on-chip of 70 min,	Au nanorods	The effect of seed age on the growth of gold nanorods was investigated in real time. As the gold-grain nanoparticle solution ages, the efficiency of the nanorods decreases.	[239]
	1 mM HAuCl_4_, 100 mM CTAB, 10 mM acetylacetone (acac), 100 mM CTAB, 100 mM and 0.025–0.1 mM AgNO_3_	30 °C	Continuous flow microreactor with rotating tube processor (RTP) connecting serially with a Narrow channel processor (NCP).	RTP flow rate: 40 mL/min, NCP flow rate: 10 mL/min,	Au nanorods	Development of method for production of AuNRs at room temperature without using seeds, applying acetylacetone as the reductant.	[240]
	10 mL 0.5 mM–6 mM HAuCl_4_, 0.5 mM–12 mM ascorbic acid	pH from 10 to 11.8 (ascorbic acid)	Continuous flow millifluidic setup	the injection rate: 5 mL/min, flow rates: 30 mL/min and 240 mL/min	3 to 25 nm	Obtained biocompatible gold nanoparticles of controlled size at ambient temperature. The size of the gold nanoparticles was controlled by adjusting the initial pH of the ascorbic acid or the flow rate of the solutions.	[241]
	3.8 mM NaBH4, 1 mM HAuCl_4,_ 20 mM tetradecyltrimethylammonium bromide	100 °C	Segmented flow microreactor	residence time: 10 s, flow rate: 27, 273 and 300 μL/min	toluene: particles with a size of 3.8 ± 0.3 nm; air 2.8 ± 0.2 nm; silicone oil 7.8 ± 6.5 and 15.5 ± 3.1 nm	The sliding speed between the two fluids and the internal mixing in the continuous phase determines the nature of the particle size distribution. Reducing the axial dispersion has less impact on particle growth and therefore on the particle size distribution.	[242]
Pt	5.4 mmol/L H_2_PtCl_6_ × 6H_2_O, sodium hydroxide, PVP, NaOH	110–140 °C	X-type micromixer	flow rate: 0.1 0.4, 0.8 mL/min, Reynolds numbers less than 15	1–4 nm	The particle size of the Pt nanoparticles which are formed inside the microchannels can be controlled by the NaOH/Pt ratio. At a high NaOH/Pt ratio, the Pt nanoparticles have a lower size and a narrow size distribution. At a low NaOH/Pt ratio, particle size increases, also the particle size distribution increases.	[243]
	1.0 × 10^−1^ wt % heptane solution with H_2_PtCl_6_· 6H_2_O, 6.9 wt %Brij L4, 1.9 wt % water, and 8.7 × 10^−1^ wt % methanol, heptane solution with 2.6 × 10^−2^ wt % NaBH_4_, 6.9 wt % Brij L4, 1.9 wt % sodium hydroxide solution and 8.7 × 10^−1^ wt % methanol	pH 11	PTFE tube	flow rate: 1.0 × 10^−4^ to 1.0 × 10^−2^ m s^−1^, channel length = 2000 mm, ID = 1.0 mm	Nanosheets, size1247 ± 228 and 1.4 ± 0.2 nm	Dispersible platinum nanosheets (PtNS) were synthesized in TRAP solution in a microreactor. Due to the high dispersibility resulting from the lateral fusion of nanoplatelets, PtNSs show higher catalytic activity in the reduction of 4-nitrophenol to 4-aminophenol.	[244]
	1.93/1.125 mmol H_2_PtCl_6_· 6H_2_O, 0.5-M-NaOH/EG solution, 40 mmol oleic acid, 40 mmol oleylamine and 50 mL hexadecene	170 °C–200 °C,	Biphasic micro tube reactor (MTR)	reaction time of 90 s	2 nm	With the increase in the reaction temperature, the rate constant k decreases simultaneously from 6.5 × 10^−5^/s at 160 °C to 3.2 × 10^−6^ /s at 170 °C.	[245]
Pd	7.42 mM K_2_PdCl_4_	180 °C	PFA tube	ID = 1.0 mm, flow rate: 0.2 mL/min, residence time d 10 do 150 cm	~13.0 ± 3.5 nm	Preparation of surfactant-free Pd nanocrystals (NC) uniformly loaded on N-doped porous carbon in a microfluidic system. Pd/N-carbon composites show outstanding activity for the formic acid electrochemical oxidation.	[246]
	8.3 mM of β-d-Glucose, 36.9 mg of soluble starch and 1.78 mL of H_2_PdCl_4_, 0.5 M NaOH	pH 5.5, 60 °C	Tygon^®^ tubing	residence time: 10 min, flow rate: 132 mL/h,	7.2 ± 21.1 nm and 4.40 ± 1.68	The ecological synthesis of palladium nanoparticles was carried out using polysaccharides as reducing agents in a microreactor. Stable PdNP suspensions with small and well-dispersed particles were obtained. Nanoparticles synthesized in a microreactor showed high catalytic activity in the degradation of 4-nitrophenol.	[247]
	50 mM Palladium(II) acetate in toluene, oleylamine, methanol and hexanol	100 °C	Si-Pyrex microreactor	~270 µm deep, 700 µm wide, and 80 cm long, flow rate: 1 mL/min	1.0 nm and 3.0 nm	Synthesis of colloidal Pd nanoparticles in the presence of oleylamine (OLA) and trioctylphosphine (TOP) ligands using in situ SAXS and XAFS in microflow. Protective ligands slow down growth, resulting in continuous nucleation of nanoparticles. The final size of the nanoparticles was influenced by the binding strength of the ligands. Strongly binding TOP ligands led to synthesis of Pd nanoparticles with a size of 1 nm with a narrow size distribution (±20%), while OLA led to particles with a size of 3 nm (±20%).	[248]
	Na_2_PdCl_4_ in mixtures of water, ethylene glycol, PVP and KBr	160 °C and 190 °C	Silicon/Pyrex microreactors	V: 100 μL, pressure: 0.8 MPa, gas/liquid volume flow ratios were in the range of 0.8 to 3.5.	4 nm Pd rod-shaped nanostructures	Synthesis of Pd rod-shaped nanostructures in laminar and segmented flow. The use of air as a segmentation gas resulted in an anisotropic increase in Pd, and increased temperatures (160 °C and 190 °C) and pressure (0.8 MPa) shortened the synthesis time to 2 min. The resulting Pd nanorods showed high activity at moderate temperature (40 °C) and pressure (0.2 MPa) during the catalytic hydrogenation of styrene.	[216]
	Na_2_PdCl_4_, L-ascorbic acid, sunflower oil, trimethyl chitosan (TMC)	25 °C	New microfluidic platform	60 mm (W) × 20 mm (H), flow rates: 50 and 250 µL/ min	spherical 35–40 nm	Designing a microreactor for the continuous synthesis of nanoparticles of controlled sizes and shapes in drops. Trimethylchitosan (TMC)-coated palladium (Pd) nanoparticles were fabricated for biomedical applications.	[249]
	Na_2_PdCl_4_, L-ascorbic acid, KBr, PVP	80 °C	PTFE tube	PTFE tube was reduced to ca. 500 μm,	spherical ca. 5 nm, cubes/bars ca.10, 14, 18 nm		[250]

* Chemicals: metal precursor, reducing agent, stabilizer; ** Reacting conditions: reagents concentration, temperature, pH of the solution.

## Data Availability

No new data were created in this study.
